# In Silico Modeling of Structural Compatibility and Alignment Between Viral Class I Fusion Cores and Human TLR4/MD-2

**DOI:** 10.3390/ijms27125317

**Published:** 2026-06-12

**Authors:** Ralf Kircheis

**Affiliations:** Advanced Medical Solutions (AMS) Saal, 93342 Saal an der Donau, Germany; ralf.kircheis@admedsol.com; Tel.: +49-151-167-90606

**Keywords:** toll-like receptor (TLR), SARS-CoV-2 spike protein, COVID-19, HR1HR2 fusion core, influenza virus, respiratory syncytial virus (RSV), Ebola virus, molecular docking, immune hyperactivation

## Abstract

The SARS-CoV-2 spike protein has been shown to activate Toll-like receptor 4 (TLR4), yet the precise molecular structures driving recognition and subsequent activation remain poorly defined. Here, we present in silico structural alignments and molecular docking simulations indicating potential spatial compatibility between the wild-type SARS-CoV-2 HR1HR2 fusion core and the human TLR4/MD-2 heterodimer. The computational models project candidate interfaces involving salt bridges, as well as polar and non-polar interactions, with both TLR4 and MD-2 dimerization partners, suggesting a theoretical topology compatible with the dimerization of two TLR4/MD-2 heterocomplexes. Notably, similar structural compatibility was modeled for related class I fusion proteins from other highly pathogenic viruses, including SARS-CoV, MERS-CoV, influenza viruses A, B, and C, respiratory syncytial virus (RSV), and partially Ebola virus. These findings offer an exploratory computational hypothesis regarding viral–host interactions with the host innate immune system, which can trigger immune recognition or detrimental hyperactivation.

## 1. Introduction

Severe acute respiratory syndrome coronavirus 2 (SARS-CoV-2) has caused one of the largest global pandemics in recent history, accounting for nearly 780 million confirmed cases and over 7.1 million deaths worldwide by January 2026. Over the past century, a variety of devastating viral outbreaks have repeatedly threatened global healthcare systems. These include the 1918–1920 Spanish flu (50–100 million deaths), the Asian flu (1957–1958; 1–2 million deaths), and the Hong Kong flu (1968–1969; 1–4 million deaths), alongside highly lethal epidemics driven by SARS-CoV (mortality rate ~10%), MERS-CoV (mortality rate ~34%), and Ebola virus (mortality rate 40–90%) [1,2,3,4].

A defining feature of these highly pathogenic viruses is their high transmission efficiency coupled with severe pathogenicity. Although the precise mechanisms underlying cellular entry, tissue tropism, and replication cycles differ substantially among these different viruses depending on specific host receptor interactions [4,5,6,7,8], their pathological manifestations frequently converge into a shared clinical syndrome driven by immune hyperactivation. This response typically manifests as a systemic T-helper 1 (Th1)-biased hyperinflammation. While a tightly regulated inflammatory response is critical to initiate host defense mechanisms, uncontrolled immune activation can be highly detrimental to the host. Severe acute viral infections are characteristically associated with an excessive release of pro-inflammatory cytokines (including TNF-α, IL-1, and IL-6) and chemokines (including IL-8, CXCL1, CXCL2, and CCL2), directly correlating with elevated morbidity and mortality [9,10,11,12,13,14,15,16,17,18,19,20].

Although late-stage clinical manifestations diverge based on viral tropism (e.g., respiratory failure versus hemorrhagic fevers) and replication kinetics (rapid acute onset versus prolonged incubation), the early pathophysiological phases exhibit striking similarities. These initial stages typically present as generalized flu-like symptoms, including high fever, severe malaise, myalgia, and headache, which can rapidly progress to severe systemic and neurological complications such as hypotension and cognitive confusion. These clinical similarities suggest the existence of shared, core pathophysiological mechanisms among diverse highly pathogenic viruses [21,22,23,24,25,26,27], likely mediated by conserved intracellular viral replication steps or shared interactions with host cell surface receptors and signaling pathways [28]. For instance, severe cases of respiratory viral infections—including SARS-CoV, MERS-CoV, influenza viruses A, B, and C, and respiratory syncytial virus (RSV)—frequently lead to acute respiratory distress syndrome (ARDS). This life-threatening condition is characterized by a rapid-onset, widespread pulmonary inflammation, massive macrophage (M1)/Th1 cytokine release, disrupted alveolar–capillary barrier permeability, and the subsequent accumulation of protein-rich edematous fluid within the alveolar spaces. Strikingly, these pathological shifts are similar to the acute lung injury induced by severe bacterial infections, sepsis, pneumonia, or acute toxic chemical inhalation, and can be driven either by localized tissue inflammation or by systemic, blood-borne inflammatory mediators [29,30,31,32].

Beyond localized respiratory syndromes, this systemic hyperactivation of the innate immune system is characteristic of a broader spectrum of acute, highly pathogenic viral infections, including Ebola virus disease and severe Dengue fever. A hallmark of these infections is a profound, pro-inflammatory hypercytokinemia, commonly termed a cytokine storm [9,10,11,12,13,14,15,16,17,18,19,20,27,28,33,34]. At the molecular level, this host–virus interaction is largely mediated by host pattern recognition receptors (PRRs) that detect viral pathogen-associated molecular patterns (PAMPs) and trigger intrinsic danger signaling cascades [35,36]. Among these, Toll-like receptor 4 (TLR4) is known as a central driver of immune hyperactivation. While classically known as the sensor for bacterial lipopolysaccharide (LPS), TLR4 activation has been increasingly implicated or hypothesized in the pathogenesis of various enveloped viruses, including SARS-CoV, MERS-CoV, influenza viruses, RSV, Ebola virus, and Dengue virus [36,37,38,39]. Given the expanding list of viral structures identified as PRR agonists, identifying conserved structural modalities among these different viral components is essential for developing targeted therapeutic interventions capable of controlling detrimental immune hyperactivation.

To identify these shared structural determinants, it is critical to evaluate essential steps in the viral replication cycle that are conserved across distinct viral families. Membrane fusion—the critical mechanism enabling viral entry via the fusion of the viral envelope with the host cellular membrane—represents such a universally conserved process. This step is mediated by dedicated macromolecular fusion complexes utilized by a wide array of enveloped viruses, including highly pathogenic coronaviruses (*Coronaviridae*), influenza viruses A, B, and C (*Orthomyxoviridae*), respiratory syncytial virus (*Paramyxoviridae*), and Ebola virus (*Filoviridae*). A defining feature of these diverse virus families is their reliance on class I viral fusion proteins to mediate host cell entry. Prominent examples include the spike (S) protein of coronaviruses, hemagglutinin (HA) in influenza viruses, the fusion (F) glycoprotein in RSV, and the glycoprotein 2 (GP2) subunit in Ebola virus. Despite originating from phylogenetically distinct families, these class I fusion proteins share highly conserved structural similarities, i.e., they exist as homotrimers in both their metastable pre-fusion and highly stable post-fusion conformations [40].

In the native pre-fusion state, the fusion subunit is typically clamped and kinetically trapped by a receptor-binding subunit. Activation of the fusion-competent protein requires a specific trigger, such as host cell receptor binding, proteolytic cleavage, or exposure to the acidic environment of the endosome. This triggering event releases the structural clamp, inducing a massive conformational rearrangement that converts the native protein into a transient, membrane-embedded pre-hairpin intermediate. This intermediate subsequently collapses into a highly stable trimer-of-hairpins conformation, characterized by a central, N-terminal trimeric alpha-helical coiled-coil surrounded antiparallelly by three C-terminal helices. This structural arrangement forms a canonical six-helix bundle (6HB). The primary biophysical function of the 6HB is to mechanically bring the hydrophobic viral envelope and the host cellular membrane into close apposition, thereby overcoming the hydration barrier to initiate lipid bilayer fusion and subsequent viral entry. Because many of these conformational transitions are strictly dependent on low pH, endosomes constitute a primary anatomical site for virus–cell membrane fusion [40].

Importantly, this fusion process not only represents a critical step for viral genome delivery but also serves as a potent driver of host pathophysiological responses. Emerging evidence indicates that several class I fusion proteins act as direct exogenous agonists of host pattern recognition receptors, particularly TLR4. Specifically, the coronavirus spike protein, the RSV F-protein, and the Ebola virus GP2 subunit have all been demonstrated to trigger TLR4 activation [35,36,37,38,39,41,42,43,44]. To elucidate the shared structural motifs underlying this receptor engagement, the present study analyzed in silico the binding architecture and structural complementarity between these diverse class I viral fusion complexes and the human TLR4/MD-2 receptor heterodimer. Through comparative structural alignment and molecular docking simulations, we investigate whether these viral fusion domains share a conserved spatial mechanism capable of driving TLR4/MD-2 dimerization and subsequent downstream inflammatory signaling.

The SARS-CoV-2 spike (S) glycoprotein is expressed on the viral envelope as a homotrimer, with each protomer comprising a large extracellular ectodomain, a single-pass transmembrane anchor, and a short C-terminal intracellular tail. The ectodomain comprises the receptor-binding S1 subunit and the membrane-fusing S2 subunit. Host cell attachment is initiated when the receptor-binding domain (RBD) within the S1 subunit binds to the host cell surface-exposed angiotensin-converting enzyme 2 (ACE2) receptor with high affinity. Following binding to the ACE2 receptor, the S2 subunit—upon proteolytic processing at the S2’ site by the host serine protease TMPRSS2—mediates the fusion of the viral and host cellular membranes [45]. Structurally, the S2 subunit contains a hydrophobic fusion peptide, two highly conserved heptad repeat regions (HR1 and HR2), the TMPRSS2 cleavage site, and the transmembrane (TM) domain [46].

High-affinity ACE2 binding of wild-type SARS-CoV-2 followed by TMPRSS2 cleavage, particularly in ACE2- and TMPRSS2-rich tissues such as alveolar epithelial cells, has been considered the main trigger mechanism for high viral replication correlating with pathophysiological consequences [14,15,45,46]. The emergence of recent variants of concern (VoCs) has challenged this strict correlation. Notably, highly transmissible variants such as the Omicron strains [47] exhibit a distinct uncoupling between infection/transmission efficiency and severe clinical pathophysiology. Despite their significantly enhanced transmissibility, Omicron lineages cause markedly attenuated clinical severity compared to the ancestral wild-type virus and early VoCs (e.g., Delta) [48,49,50,51,52].

This uncoupling highlights the biological importance of the alternative endosomal entry pathway. In TMPRSS2-deficient but cathepsin L-rich environments, SARS-CoV-2 undergoes internalization via clathrin-mediated endocytosis, where the endosomal cysteine protease cathepsin L by proteolytic cleavage activates the S2 subunit [53]. This endosomal entry route is particularly predominant in endothelial cells and cells of the innate immune system [54]. Beyond driving viral entry, shed or structural components of SARS-CoV-2, most notably the spike protein, act as potent pathogen-associated molecular patterns (PAMPs). SARS-CoV-2 spike protein was shown to activate host pattern recognition receptors, particularly Toll-like receptors (TLRs) TLR2 and TLR4, triggering robust downstream activation of the canonical nuclear factor kappa B (NF-κB; p50/p65) signaling cascade [28,54]. Importantly, the cellular expression profiles of the different TLRs are highly tissue- and cell-type-specific. While alveolar epithelial cells express minimal baseline levels of TLRs, innate immune cells (such as macrophages and dendritic cells) and endothelial cells exhibit high surface densities of TLR4. Consequently, the physical interaction of the SARS-CoV-2 spike protein with TLR4 on cells of the innate immune system and endothelial cells represents a primary mechanism of PAMP-mediated pathogenesis. The resulting activation of the TLR-dependent MyD88 pathway drives the nuclear translocation of NF-κB, culminating in the transcriptional hyperactivation and systemic release of classical pro-inflammatory cytokines, including TNF-α, IL-1, IL-6, and IL-12 [14,35,54].

To elucidate the shared structural motifs triggering TLR4 recognition and activation, the present study analyzed in silico the binding architecture and structural complementarity between diverse class I viral fusion core complexes and the human TLR4/MD-2 receptor heterodimer.

## 2. Results

### 2.1. Molecular Docking Indicates Spatial Compatibility of the SARS-CoV-2 HR1HR2 Fusion Core and Human TLR4/MD-2 Compatible with Dimerization of Two TLR4/MD-2 Heterocomplexes

SARS-CoV-2 has been demonstrated to act as an activator of TLR4. Specifically, the full-length SARS-CoV-2 spike (S) protein trimer was shown to activate TLR4 [41,42,43,44,54,55]. In this regard, ligand-induced dimerization of two TLR4/MD-2 heterodimers is known to be a strict prerequisite for activation of TLR4, which subsequently recruits the downstream intracellular adapter proteins Myeloid Differentiation Primary Response 88 (MyD88) and/or TIR-domain-containing adapter-inducing interferon-beta (TRIF) to initiate canonical inflammatory signaling cascades [56,57]. However, the native S trimer is a large, bulky macromolecular assembly primarily anchored to the viral envelope to mediate high-affinity binding to cellular ACE2 [45,46]. Following receptor binding, the S1 subunit is proteolytically cleaved off, leaving the remaining S2 subunit to undergo extensive conformational changes required to drive viral–host membrane fusion [46]. This spatial constraint implies that while the full-length S trimer likely interacts predominantly with surface-expressed TLR4 at the plasma membrane, processed core fusion intermediates may engage the TLR4 receptor in distinct cellular compartments.

To address this mechanism, we performed in silico molecular docking simulations to evaluate whether the fusion-active six-helix bundle (6HB) derived from the S2 subunit—represented by the cryo-EM structure of the SARS-CoV-2 HR1HR2 fusion core complex (PDB 8CZI) [58]—could directly bind the human TLR4/MD-2 heterodimer (PDB 3FXI) [59] and structurally facilitate the assembly of a dimeric active TLR4/MD-2 complex. Docking simulations calculated by HDOCK [60] yielded highly favorable thermodynamic binding profiles. The calculated docking scores were consistently below the biological relevance threshold of −200, coupled with high empirical confidence scores exceeding 0.8. The structural parameters, free energy approximations, and scoring characteristics of the top 10 ranked binding orientations are detailed in Table 1.

**Figure 1:**
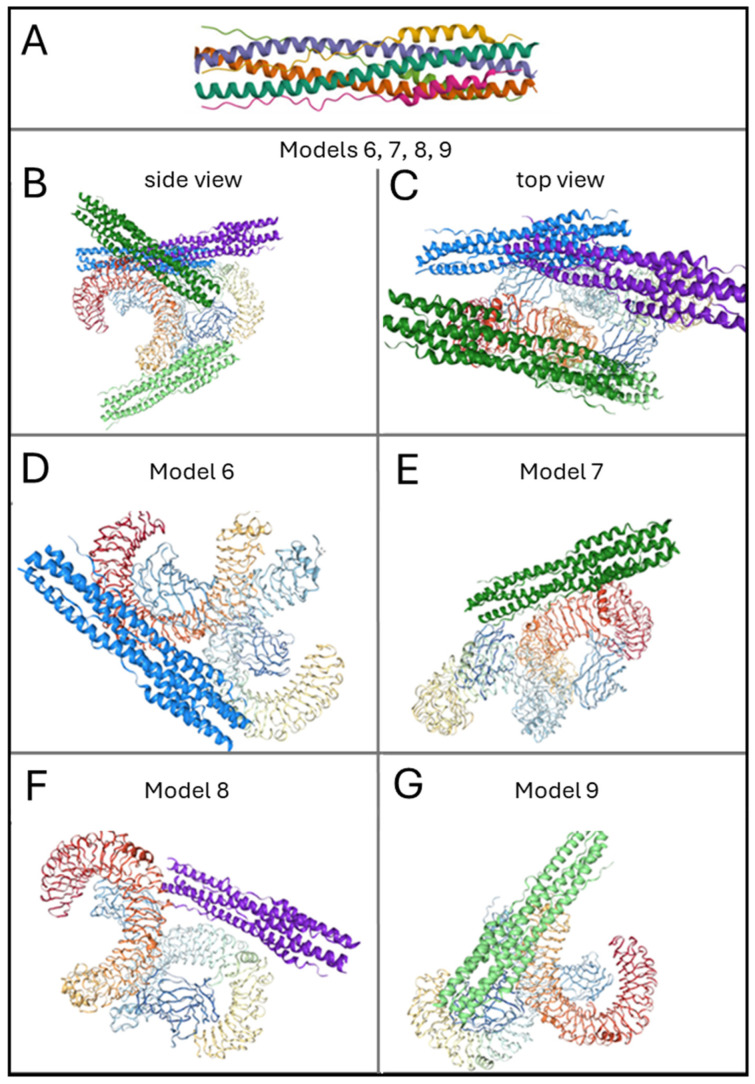
(**A**) Cryo-EM structure of the SARS-CoV-2 HR1HR2 fusion core complex with an extended HR2 domain (PDB 8CZI), with distinct colors distinguishing the three individual HR1 (blue, red, dark green) and HR2 chains (yellow, light green, magenta) within the trimer. (**B**–**G**) In silico molecular docking models of the SARS-CoV-2 HR1HR2 fusion core complex (PDB 8CZI) complexed with human TLR4/MD-2 dimers (PDB 3FXI) generated via HDOCK. Four of the top ten ranked predictive models (Models 6, 7, 8, and 9) structurally accommodated the dimerization of the TLR4/MD-2 complex mediated by the viral HR1HR2 fusion core. Binding architecture of PDB 8CZI targeting PDB 3FXI is displayed from different perspectives across all four models, including a side view (**B**) and a top-down view (**C**), as well as individual structural depictions for each specific dimerization model (**D**–**G**).

Molecular docking simulations confirmed that the binding of the SARS-CoV-2 HR1HR2 fusion core complex (PDB 8CZI; Figure 1A) to the human TLR4/MD-2 heterodimer (PDB 3FXI) structurally supports receptor dimerization of two TLR4/MD-2 heterocomplexes in four out of the top ten (4/10) ranked predictive models (Models 6, 7, 8, and 9) (Figure 1B–G).

To validate the consistency of this interaction, docking simulations were repeated using alternative PDB-derived structural repositories of the SARS-CoV-2 HR1HR2 fusion core, specifically PDB 7RZQ [61], PDB 2BEZ [62], and PDB 6M1V [63]. Intriguingly, across all four independent structural variants of the SARS-CoV-2 HR1HR2 fusion core, exactly four out of the top ten predictive models (4/10) consistently facilitated the dimerization of the two TLR4/MD-2 heterodimers. Representative docking configurations for PDB 7RZQ (Models 1, 2, 4, and 7) are shown in Figure S1.

**Figure 2:**
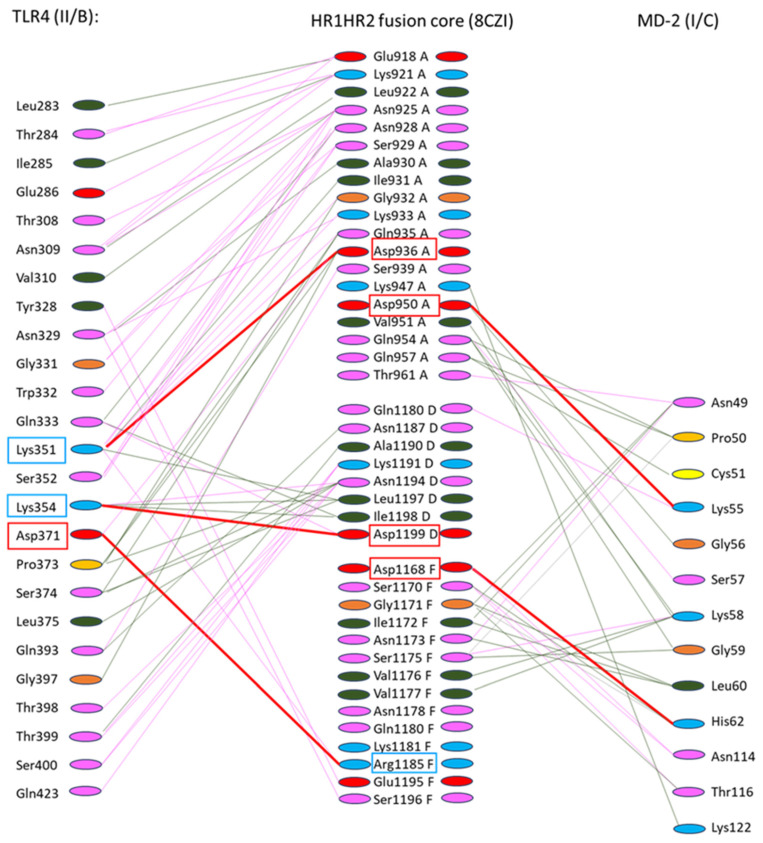
Molecular interface interactions of the SARS-CoV-2 HR1HR2 fusion core complex within Model 6, highlighting contacts with the TLR4 subunit of the dimerization partner TLR4/MD-2 (II) and the MD-2 subunit of partner TLR4/MD-2 (I). Residue color coding: blue, positively charged; red, negatively charged; pink, polar; dark green, non-polar, brown—glycine, orange—proline; yellow—cysteine. Inter-residue interaction types: thick red lines, salt bridges; thin pink lines, hydrogen bonds; thin gray lines, hydrophobic and van der Waals interactions.

To elucidate the spatial mechanism driving receptor assembly, the specific interfacial amino acid contacts and binding energies between the SARS-CoV-2 HR1HR2 fusion core complex (PDB 8CZI) and the TLR4/MD-2 heterodimers were analyzed. The HDOCK algorithm mapped an extensive network comprising distinct ionic bonds, hydrogen bonds, and non-polar interactions across the macromolecular interface. Figure 2 illustrates these molecular interactions for Model 6, which represents the dimerization mode with the most favorable thermodynamic docking score. In this configuration, the SARS-CoV-2 HR1HR2 fusion core complex simultaneously establishes contacts with the TLR4 subunit of the second receptor partner (TLR4/MD-2 [II]) and the MD-2 subunit of the first receptor partner (TLR4/MD-2 [I]). Several prominent salt bridges were identified within these dual interfaces. Within the viral core–TLR4 (II) interface, residues Lys921, Asp936, Asp1199, and Arg1185 of the SARS-CoV-2 fusion core build salt bridges with Glu286, Lys351, Lys354, and Asp371 of the TLR4 subunit, respectively (Figure 2). Concurrently, the viral core–MD-2 (I) interface was stabilized by salt bridges formed between Asp950 and Asp1168 of the SARS-CoV-2 fusion core and residues Lys55 and His62 of the MD-2 subunit, respectively (Figure 2). The lower frequency of salt bridges established with the MD-2 component relative to the TLR4 subunit suggests that the viral core–MD-2 interface may represent the rate-limiting step in receptor dimerization, requiring cooperative stabilization by the surrounding network of polar and non-polar contacts.

**Figure 3:**
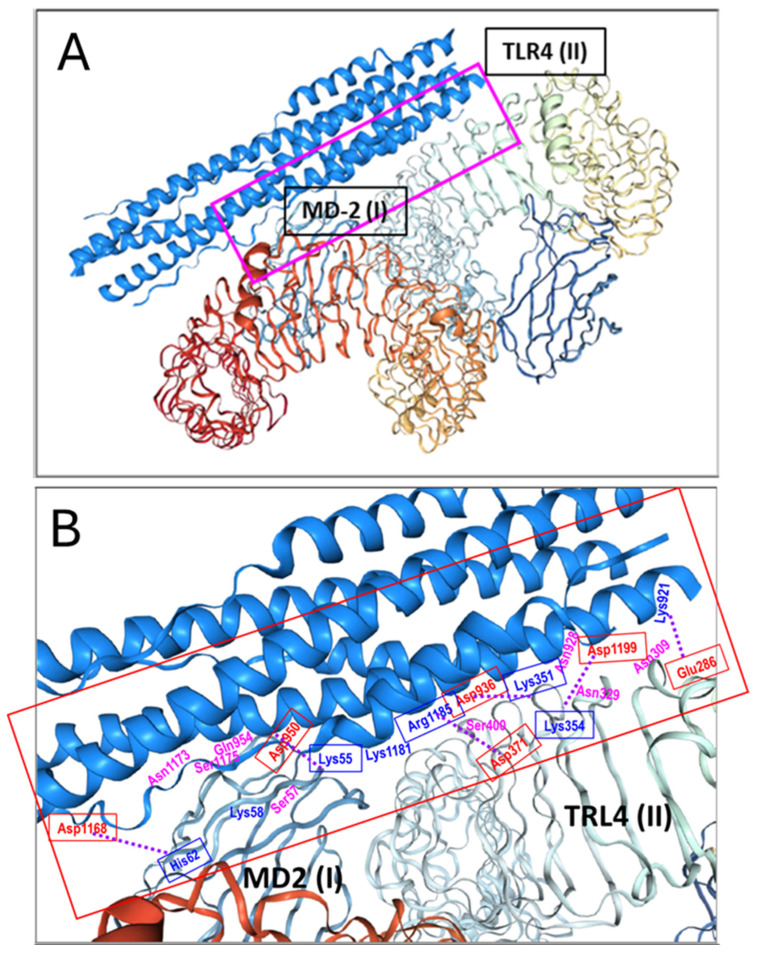
Spatial overview and interface magnification of the molecular interactions between the SARS-CoV-2 HR1HR2 fusion core and the TLR4/MD-2 heterodimers. (**A**) Global docking architecture generated via HDOCK (cartoon representation). (**B**) Magnified view of the interaction interface highlighting key ionic and polar contact residues. Residue color coding: blue, positively charged; red, negatively charged; pink, polar. Dotted lines represent stabilizing salt bridges.

The spatial arrangement of the SARS-CoV-2 HR1HR2 fusion core complex bound to the TLR4/MD-2 heterodimers is illustrated in Figure 3. A high-magnification view of the core interaction interface illustrates the distinct ionic and polar networks, highlighting the key contributing residues (Figure 3B).

**Figure 4:**
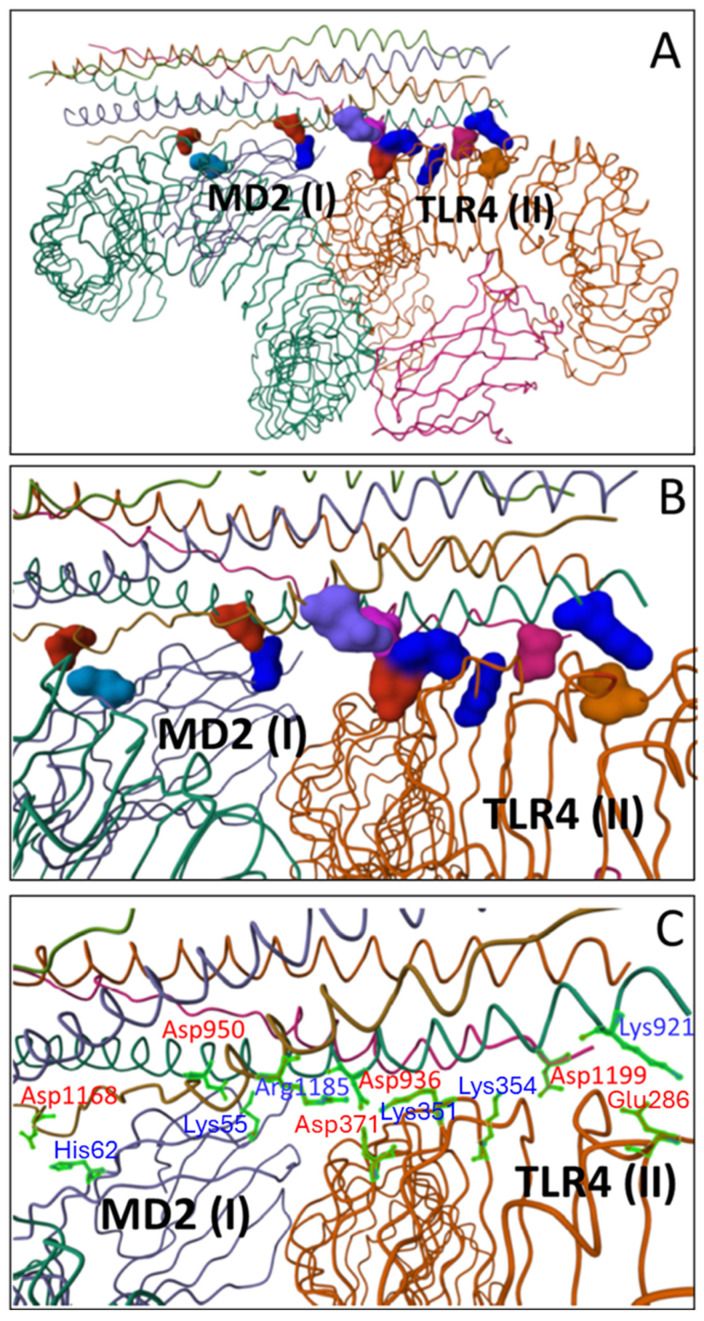
Spatial coordination of interface residues participating in salt-bridge formations between the SARS-CoV-2 HR1HR2 fusion core and the TLR4/MD-2 heterocomplex (displayed sequentially from right to left). Viral fusion core interactions with the TLR4 subunit of partner II involve Lys921–Glu286, Asp1199–Lys354, Asp936–Lys351, and Arg1185–Asp371. Viral core interactions with the MD-2 subunit of partner I comprise Asp950–Lys55 and Asp1168–His62. Interfacial residues are rendered using Mol* as molecular surfaces (**A**,**B**) or ball-and-stick models (**C**). Residue color coding for electrostatic surface potentials: positively charged (Lys, blue; Arg, purple; His, turquoise); negatively charged (Asp, red/pink; Glu, orange).

The spatial distribution of charged amino acid residues mediating these salt bridges across the macromolecular boundary is detailed in Figure 4. The structural models map the precise topology of the two salt bridges established with the MD-2 subunit of dimerization partner I, alongside the four salt bridges targeting the TLR4 subunit of dimerization partner II, visualized via molecular surface and ball-and-stick representations (Figure 4A–C).

**Figure 5:**
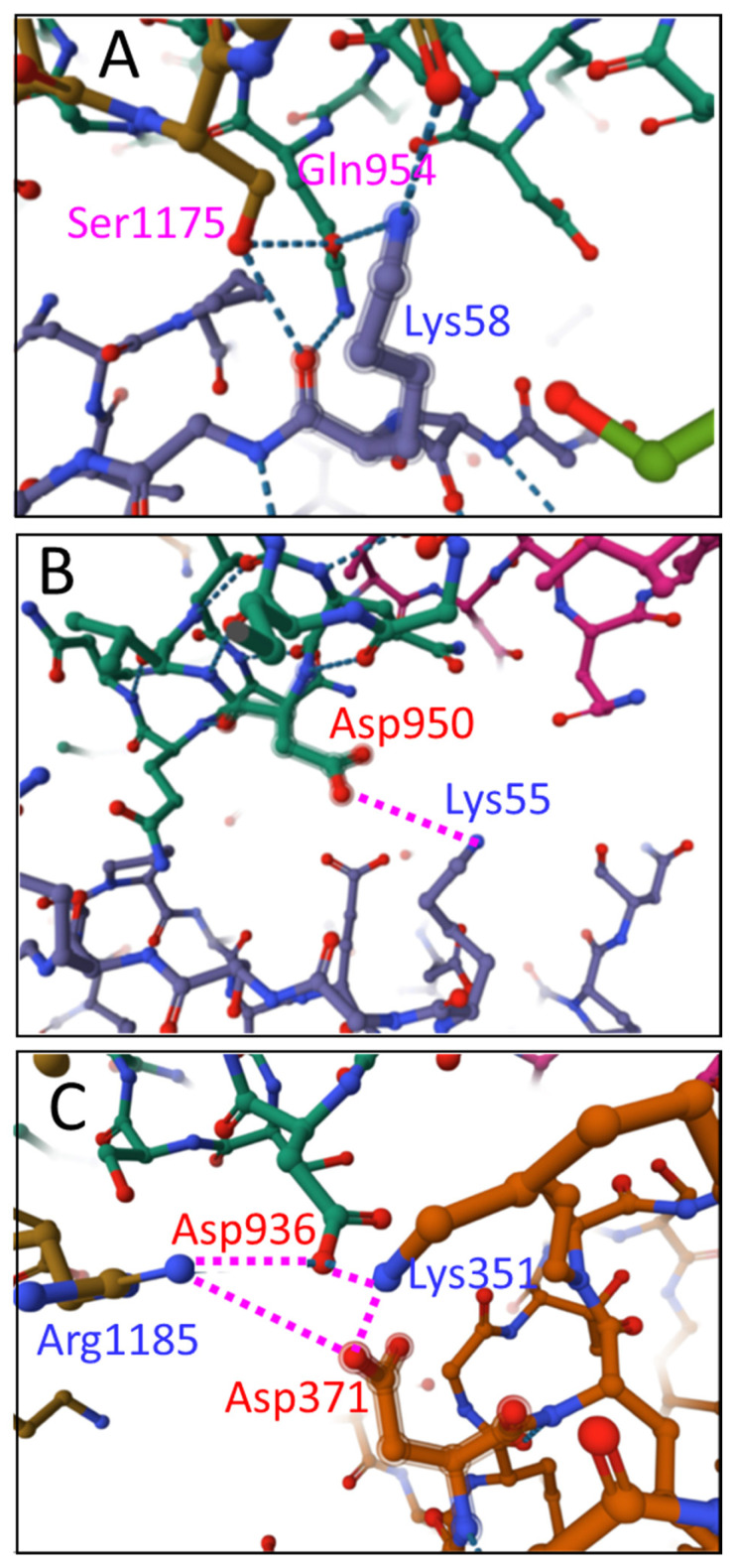
High-resolution analysis of three selected molecular interaction areas at the binding interface between the SARS-CoV-2 HR1HR2 fusion core complex and TLR4/MD-2. (**A**) Local network involving Ser1175 (fusion core chain F, brown), Gln954 (fusion core chain A, green), and Lys58 (MD-2 [I], blue, showing oxygen and NH_3_^+^ groups); blue dotted lines indicate hydrogen bonds. (**B**) Interaction between Asp950 (fusion core chain A, green) and Lys55 (MD-2 [I], blue); pink dotted lines indicate salt bridges. (**C**) Complex electrostatic network involving Arg1185 (fusion core chain F, brown, left), Asp936 (fusion core chain A, green), Lys351 (TLR4 [II], red, right), and Asp371 (TLR4 [II], red, below); pink dotted lines indicate salt bridges.

To further evaluate the local topography of these binding interfaces, three distinct regions of high structural complementarity between the SARS-CoV-2 HR1HR2 fusion core and the TLR4/MD-2 complex were zoomed, representing two contact areas targeting MD-2 (I) (Figure 5A,B) and one targeting TLR4 (II) (Figure 5C).

The docking models depicted in Figure 3, Figure 4 and Figure 5 indicate that a cluster of solvent-exposed, charged amino acids (Lys921, Asp936, Asp950, Asp1168, Arg1185, and Asp1199), alongside specific polar residues (Ser1175 and Gln154), are crucially involved in mediating the interfaces with the two TLR4/MD-2 heterodimers, respectively. Because these residues reside in highly exposed positions on the outer surface of the trimeric SARS-CoV-2 HR1HR2 post-fusion bundle, they are sterically accessible to initiate receptor contacts.

To evaluate the functional relevance of these specific positions, molecular docking simulations were performed using the single amino acid exchange variant D936Y (PDB 7RZR, [61]). The D936Y substitution significantly impaired TLR4/MD-2 dimerization potential, reducing the frequency of productive homodimerization configurations to only two out of the top ten predictive models (2/10), compared to four out of ten (4/10) for the wild-type core (Figure S2).

To further validate these observations across evolutionary lineages, docking simulations were performed using multiple mutational variants of the SARS-CoV-2 HR1HR2 fusion complex. First, an intermediate spike variant harboring the single Omicron-associated substitution Q954H (PDB 8VYA, [64]) was tested. This variant maintained a wild-type-like dimerization profile, with four out of the top ten ranked binding models (Models 2, 4, 5, and 8) enabling TLR4/MD-2 homodimerization (Figure S3), consistent with the ancestral repositories (PDB 8CZI and 7RZQ).

In contrast, docking simulations using the Omicron HR1HR2 fusion core complex (PDB 7TIK [65])—which harbors three amino acid residue substitutions, i.e., Q954H, N969K, and L981F—completely failed to support receptor homodimerization among the premier orientations, resulting in a dimerization frequency of zero out of ten (0/10) (Figure S4).

To isolate the specific molecular driver behind this loss of function, we analyzed a single-mutant fusion core complex displaying only the Omicron-typical N969K substitution (PDB 8FA1, [65]). Intriguingly, the N969K single mutant demonstrated a severe reduction in receptor assembly, with only one out of the top ten models (1/10) capable of inducing TLR4/MD-2 dimerization (Figure S5).

Taken together, these mutational docking data demonstrate distinct structural effects of different amino acid substitutions in the viral post-fusion bundle (Table 2). While the single Q954H mutation does not alter receptor dimerization potential, the three amino acid residue substitutions characteristic for Omicron abolish this capacity within the top-ranked binding models. Notably, the single N969K substitution accounts for the most part of this functional impairment. These in silico findings align with literature describing the structural adjustments in the Omicron fusion core. While Q954H has been shown to optimize viral fusion kinetics by stabilizing local intra-trimer interactions between the HR1 and HR2 domains [64], the adjacent N969K mutation induces a major structural displacement of the heptad repeat 2 (HR2) peptide backbone within the post-fusion bundle. This local backbone remodeling not only explains the observed drop in TLR4/MD-2 dimerization potency but also correlates with the known resistance of Omicron lineages to fusion-entry peptide inhibitors modeled on the ancestral Wuhan-Hu-1 sequence [65].

### 2.2. The SARS-CoV-2 HR1HR2 Fusion Core Complex Shares Structural Homology with Fusion Complexes of Diverse Highly Pathogenic Enveloped Viruses

**Figure 6:**
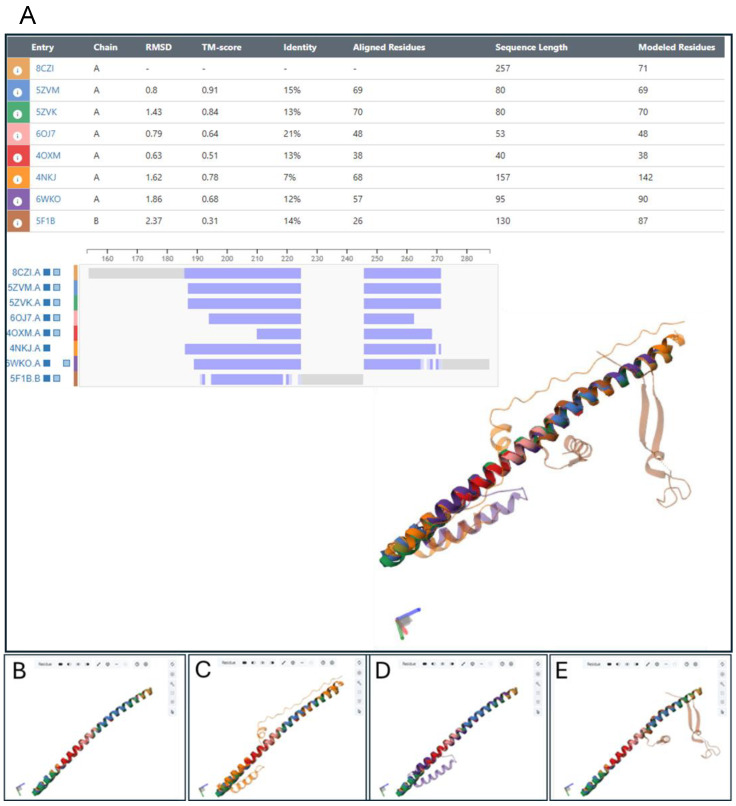
Pairwise backbone structural alignments of diverse viral fusion proteins against the SARS-CoV-2 spike HR1HR2 fusion core complex. (**A**) Structural overlay showing monomers of the SARS-CoV-2 spike HR1HR2 fusion core (PDB 8CZI), SARS-CoV spike HR1 motif (PDB 5ZVM), MERS-CoV spike HR1 motif (PDB 5ZVK), respiratory syncytial virus (RSV) fusion glycoprotein N-terminal heptad repeat domain (PDB 6OJ7), central coiled-coil from influenza A virus hemagglutinin HA2 (PDB 4OXM), influenza B virus hemagglutinin at membrane fusion pH (PDB 4NKJ), influenza C virus hemagglutinin-esterase-fusion (HEF2) intermediate (PDB 6WKO), and the Ebola virus glycoprotein 2 subunit (GP2; PDB 5F1B, chain B). (**B**) Structural overlay demonstrating high structural homology between PDB 8CZI and entries 5ZVM, 5ZVK, 6OJ7, and 4OXM. (**C**) Alignment showing high structural similarity with PDB 4NKJ. (**D**) Alignment showing structural similarity with PDB 6WKO. (**E**) Alignment showing localized, partial structural convergence with PDB 5F1B, chain B. For visual clarity, only monomeric subunits of the respective fusion complexes are displayed. Pairwise alignments against the complete trimeric assembly of PDB 8CZI and detailed amino acid sequences are shown in Figure S6.

To explore the evolutionary and structural conservation of the viral fusion machinery, the cryo-EM structure of the SARS-CoV-2 HR1HR2 fusion core complex (PDB 8CZI) was systematically compared against corresponding post-fusion architectures from various highly pathogenic enveloped viruses using the RCSB PDB pairwise structural alignment tool. The comparative panel included the SARS-CoV spike HR1 motif (PDB 5ZVM) [66], the MERS-CoV spike HR1 motif (PDB 5ZVK) [66], the respiratory syncytial virus (RSV) fusion glycoprotein N-terminal heptad repeat domain (PDB 6OJ7) [67], the central coiled-coil from influenza A virus hemagglutinin HA2 (PDB 4OXM) [68], the influenza B virus hemagglutinin at membrane fusion pH (PDB 4NKJ) [69], the influenza C virus hemagglutinin-esterase-fusion (HEF2) intermediate (PDB 6WKO) [70], and the Ebola virus glycoprotein 2 subunit (GP2; PDB 5F1B, chain A) [71] (Figure 6A).

Pairwise backbone alignments revealed a high degree of spatial conservation across the various examined viruses. Strong overall structural homology was observed between the SARS-CoV-2 core (PDB 8CZI) and entries 5ZVM, 5ZVK, 6OJ7, and 4OXM (Figure 6B). Pronounced structural similarity was likewise maintained with the fusion intermediates of influenza B (PDB 4NKJ; Figure 6C) and influenza C (PDB 6WKO; Figure 6D), while a localized, partial structural alignment was mapped for the Ebola virus GP2 subunit (PDB 5F1B, chain B; Figure 6E).

At the secondary and tertiary structural levels, the central alpha-helical coiled-coil domain represents the primary anchor of spatial alignment across all evaluated virues. Within the *Coronaviridae* family, the post-fusion bundles of SARS-CoV, SARS-CoV-2, and MERS-CoV aligned with exceptional precision, yielding high Template Modeling scores (TM-scores of more than 0.8) and low root-mean-square deviations (RMSD less than 2 Å). Intriguingly, this structural conservation extended to phylogenetically unrelated, highly pathogenic airborne viruses. The fusion cores of RSV as well as influenza viruses A, B, and C displayed significant structural alignment to the SARS-CoV-2 core, characterized by biologically relevant TM-scores (>0.5) and low coordinate variances (RMSD less than 2 Å). Even the fusion core of the distinct filovirus, Ebola virus, exhibited notable structural convergence within a restricted segment of its central coiled-coil architecture (Figure 6E). This widespread spatial conservation likely reflects the shared evolutionary and biophysical constraints governing class I viral fusion proteins, which require a uniform helical bundle topology to drive host–viral membrane fusion.

### 2.3. Molecular Dockings Indicate Spatial Compatibility with Dimerization of TLR4/MD-2 Is Shared by Diverse Viral Fusion Core Complexes

To determine whether the observed structural homologies could theoretically translate into shared pathophysiological mechanisms, the core fusion proteins of the selected highly pathogenic viruses were systematically evaluated for their molecular docking profiles and structural capability to induce dimerization of human TLR4/MD-2 heterodimers. Structural entries for SARS-CoV (PDB 5ZVM) [66], MERS-CoV (PDB 5ZVK) [66], RSV (PDB 6OJ7) [67], influenza A (PDB 4OXM) [68], influenza B (PDB 4NKJ) [69], influenza C (PDB 6WKO) [70], and Ebola virus (GP2, PDB 5F1B, chain B) [71] were analyzed using molecular docking simulations utilizing the HDOCK protocol. The comprehensive thermodynamic calculations and spatial sampling metrics are summarized in Table 3 and Table 4, with extended structural orientations and individual docking topologies detailed in Figures S6 and S7A–H. All examined viral fusion structures exhibited high binding affinities for the human TLR4/MD-2 complex, characterized by calculated docking scores consistently below the biological relevance threshold of −200 and robust confidence scores exceeding 0.8. Notably, this computer-assisted screening demonstrated that the capacity to facilitate host TLR4/MD-2 receptor assembly may be highly conserved across phylogenetically diverse viruses. For every virus evaluated, at least one—and, in most cases, multiple—of the top ten ranked thermodynamic binding models structurally accommodated the dimerization of two TLR4/MD-2 heterodimers (Table 3 and Table 4).

Intriguingly, the filovirus entry mediator, Ebola virus GP2 (PDB 5F1B, chain B), yielded a distinct interaction profile: it presented exactly one productive receptor dimerization configuration within the premier cluster (Model 1). However, this specific orientation was stabilized by an exceptionally favorable binding energy, manifested by a docking score of −283. In comparison, the functional receptor-dimerization poses identified for all other airborne and respiratory viral core complexes clustered within a slightly lower, yet highly favorable thermodynamic range between −220 and −250.

## 3. Discussion

Our in silico docking models project a structural configuration compatible with TLR4/MD-2 dimerization where the SARS-CoV-2 HR1HR2 fusion core complex can bind through multiple salt bridges, as well as polar and non-polar interactions with both TLR4 and MD-2 of two TLR4/MD-2 dimerization partners. Previous studies have demonstrated that the SARS-CoV-2 spike protein (including its trimer and subunits) activates TLR4, resulting in immune system activation [41,42,43,44,54,72]. Moreover, earlier in silico analyses indicated that the SARS-CoV-2 spike protein trimer can bind to and induce the dimerization of two TLR4/MD-2 complexes [54,73]. Dimerization of the TLR4-MD-2 complex is an essential prerequisite for TLR4 activation, which is necessary for the recruitment of intracellular adaptor protein complexes, i.e., MyD88 and TRIF, which subsequently trigger intracellular signaling pathways [56,57]. However, the SARS-CoV-2 trimer is a relatively large molecule and is likely to be present in the intercellular space or bloodstream only. After receptor binding, the S1 subunit is cleaved off, leaving the S2 subunit to participate in subsequent steps of viral replication, specifically the fusion of the virus with the cellular membrane. Notably, several studies have indicated that TLR4 can also be activated by the S1 subunit [44,72], which is expected to primarily activate TLR4 at the cell surface.

In the present in silico study, we show that the SARS-CoV-2 HR1HR2 fusion core complex (PDB: 8CZI), derived from the S2 subunit of the spike protein, can bind to the human TLR4/MD-2 complex (PDB: 3FXI) and likely facilitate the dimerization of the two TLR4/MD-2 heterodimers, as supported by docking scores of less than −220 and high confidence scores exceeding 0.8. This data introduces a new dimension to our understanding of how the SARS-CoV-2 spike protein activates the innate immune system, suggesting that, in addition to TLR4 activation on the cell membrane by the spike trimer (or its subunits), the SARS-CoV-2 HR1HR2 fusion complex may activate TLR4 signaling following internalization into the host cell.

Two primary pathways of cellular uptake for SARS-CoV-2 are recognized. In addition to the well-documented binding of the SARS-CoV-2 spike protein to its main cellular receptor, ACE2 [45,46,74], which is expressed on various cells such as alveolar lung cells, the virus can alternatively be taken up via clathrin-coated pits into endosomes, followed by proteolytic cleavage by cathepsin L. Notably, endosomal uptake predominates in TMPRSS2-negative, cathepsin L-rich cells, including innate immune and endothelial cells [75,76,77,78,79]. These cells exhibit high expression levels of various TLRs, particularly TLR4 and its heterodimerization partner, MD-2 [54]. Furthermore, TLR4 is expressed on both the cell surface, where it recognizes viral proteins prior to their cellular entry, and within endosomes [80,81].

Innate immune cells and endothelial cells are significant contributors to the pronounced innate immune hyperactivation observed in severe infections caused by SARS-CoV-2, SARS-CoV, MERS-CoV, or influenza A [4,16,82,83,84,85]. The binding to and dimerization of the TLR4-MD-2 complex, which is highly expressed on innate immune cells, some epithelial cells, and endothelial cells [54], will lead to the recruitment of the intracellular adaptor protein MyD88, activating signaling pathways such as NF-κB (p50/p65), which is crucial for the massive release of pro-inflammatory cytokines, including TNFα, IL-1, IL-6, IL-8, IL-12, and IL-18 [28]. While these cytokines are essential for initiating a protective immune response [35,36,39,82,86,87], they also serve as central drivers of the cytokine storm associated with critical stages of SARS-CoV-2, SARS-CoV, MERS-CoV, and influenza infections [4,9,10,11,12,13,14,15,16,17,18,19,20,28,39,82,83,84,85].

Signaling from TLR4 expressed on the cell membrane primarily activates pro-inflammatory cytokine and chemokine release via the MyD88-dependent pathway, resulting in NF-κB activation. In contrast, signaling from the endosomal compartment has been described to activate in first instance the MyD88-independent/TRIF-dependent pathway (Toll/interleukin-1-receptor (TIR)-domain-containing adaptor protein), leading to the phosphorylation of IRF3 (IFN-regulatory factor 3) and subsequent expression of the IFNβ gene, which is crucial for inducing an antiviral state in cells, along with late NF-κB activation [39,80,81,86,87].

This study may enhance our understanding of which viral proteins or their components can facilitate recognition by TLR4, which is vital for mounting an effective antiviral immune response but may also lead to excessive innate immune stimulation [35,39,82,86,87]. Most enveloped viruses share a common mechanism for fusion with the membranes of host cells utilizing viral fusion proteins [40,88]. Pairwise structural alignment analyses in this study revealed that the SARS-CoV-2 HR1HR2 fusion core complex exhibits a high level of structural similarity with other class I fusion complexes from highly pathogenic viruses, including SARS-CoV, MERS-CoV, influenza viruses A, B, and C, RSV, and partially even Ebola virus. Importantly, all analyzed fusion complexes from different highly pathogenic viruses demonstrated a structural capability for binding and TLR4/MD-2 dimerization.

Recognition by TLR4 is critical for initiating an effective immune response and inducing an antiviral state within the cells [5,39,80,81,86,87]. However, exceeding a certain threshold of TLR4 activation can lead to hyperactivation of innate immune cells, accompanied by activation of endothelial cells lining the capillary system, resulting in cytokine release and the phenomenon known as cytokine storm. Interestingly, the pathological symptoms associated with various highly pathogenic viral infections often share a characteristic clinical syndrome complex marked by pro-inflammatory hyperactivation of the host immune system [4,9,10,11,12,13,14,15,16,17,18,19,20,39,82,83,84,85]. Similarities in general pathophysiology, particularly during the early phase, manifest as flu-like symptoms (e.g., high fever, intense weakness, muscle pain, headache), and in severe cases, pronounced systemic and neurological symptoms such as hypotension and confusion are common among these highly pathogenic viruses. Additionally, respiratory viruses such as SARS-CoV, MERS-CoV, influenza viruses A, B, and C, and RSV are known to induce acute respiratory distress syndrome (ARDS) in severe cases. ARDS is characterized by the rapid onset of widespread inflammation and lung injury, marked by significant cytokine release, increased alveolar–capillary permeability, and the accumulation of protein-rich edema fluid in the airspaces [22,23,24,25,26,27,28,29,30,31,32,33,34,35,36,37].

In the present study, the highest structural similarity was found between SARS-CoV-2 and the two other highly pathogenic coronaviruses, SARS-CoV and MERS-CoV. Notably, significant structural similarity (despite lower amino acid identity) was also observed with other airborne highly pathogenic viruses, including RSV and influenza viruses A, B, and C. Correspondingly, the fusion complexes of these viruses exhibited a common structural capability for binding and dimerization of TLR4/MD-2 heterodimers, with highly negative docking scores ranging from −220 to −250. The binding mode, as visualized by HDOCK docking studies, resembles that of SARS-CoV-2, and can be expected to have the capability to stabilize the dimerization of two TLR4/MD-2 heterodimers. The binding interactions primarily occurred on the surfaces of TLR4 and MD-2, differing from the described binding of LPS, which fits into the hydrophobic pocket of MD-2 and the groove between MD-2 and TLR4 [56,57]. For all three highly pathogenic coronaviruses—SARS-CoV-2, SARS-CoV, and MERS-CoV—binding was distributed more broadly across the surfaces of TLR4 and MD-2. In contrast, the F-protein and the influenza HA2 exhibited a slightly different binding pattern, with the fusion proteins fitting more snugly into the TLR4-MD-2 groove, correlating with slightly higher docking scores. Notably, the Ebola virus glycoprotein GP2 demonstrated the highest similarity to the well-described binding of LPS to the TLR4/MD-2 heterodimers [56,57]. The extended fusion peptide of GP2 was found to fit into the groove between TLR4 and MD-2, which correlated with the highest docking score (−283) for the dimerization of the TLR4-MD-2 partners (see also Supplementary Figure S7A–H).

The present in silico analyses comparing structurally conserved fusion complex structures of various highly pathogenic viruses may enhance our understanding of the molecular structures involved in TLR4/MD-2 binding and dimerization. The data suggest that largely hydrophobic coiled-coil structures with exposed charged amino acid residues may provide a framework that is readily recognized by the TLR4/MD-2 heterodimer complex. The binding and dimerization potency will depend on the structural compatibility of the fusion complex with the TLR4/MD-2 complexes. The eventual outcome—whether an effective antiviral immune response is generated or a pathological pro-inflammatory innate response is induced—will largely depend on the overall amplitude of TLR4 activation and the specifics of viral tropism, replication cycle, and other pathophysiological nuances, as discussed in the following section.

The activation of TLR4 by RSV F-protein has been shown to correlate with both enhanced antiviral states and innate system hyperactivation [5,9,10,36,39,89]. The RSV fusion protein has been demonstrated to induce the secretion of pro-inflammatory cytokines via TLR4/MD-2 recognition, and RSV persisted longer in the lungs of TLR4-deficient mice compared to normal mice [89], indicating that a certain level of TLR4 activation is essential for mounting an effective antiviral immune response. Conversely, the RSV fusion protein has been shown to induce TLR4-dependent neutrophil extracellular trap formation, which exacerbates inflammatory symptoms of RSV infection in young children and infants [90]. Furthermore, RSV fusion protein-induced TLR4 signaling has been shown to require direct interaction with MD-2 and can be inhibited by TLR4 antagonists such as Rhodobacter sphaeroides lipopolysaccharide and eritoran (E5564) [91].

The activation of TLR4 by fusion proteins presents particularly dramatic implications in Ebola virus disease. TLR4 activation by the Ebola virus glycoprotein (GP) is known to play a central role in the pathogenesis of Ebola virus disease [7,8,36,39,92]. Notably, in contrast to the highly pathogenic Ebola virus (EBOV), the Reston virus (RESTV)—also a member of the Ebolavirus genus—is not pathogenic in humans and lacks the ability to stimulate TLR4 in monocyte-derived macrophages (MDMs). EBOV infection elicits a profound pro-inflammatory response via the activation of IFN regulatory factor 3 (IRF3) and NF-κB, resulting in the robust induction of type I and type III interferons (IFNs), while RESTV-infected macrophages remain non-activated [92]. Importantly, even in the absence of the Ebola virus, shed GP in the bloodstream has been shown to trigger innate immune hyperactivation in monocytes [93] and activate vascular endothelium, resulting in cytokine storms and increased vascular permeability [94]. A recent study indicated that the activation of TLR4 by shed Ebola virus glycoprotein is direct and requires the internal fusion loop but is independent of glycosylation [95].

The high binding and dimerization activity of the Ebola virus GP was also confirmed in this study using a GP2 structure (5F1B_2) derived from PDB 5F1B, which binds to its endosomal receptor Niemann-Pick C1 [71]. The internal fusion loop was observed to fit into the groove between TLR4 and MD-2, facilitating dimerization of the two partners. This, combined with the fact that shed GP is released in significant amounts during Ebola virus infection, can independently lead to hyperactivation of innate immune cells and endothelial cells, resulting in a massive systemic cytokine storm and capillary leakage syndrome throughout the infected host [7,8,93,94,95].

Severe pandemic influenza has also been characterized by Th1 and Th17 hypercytokinemia as an early host response signature [96]. In this context, TLR4 has been identified as a key mediator in the hyper-inflammation observed during influenza A virus (IAV) infections, which are often accompanied by secondary bacterial infections such as Staphylococcus aureus [97]. IAV or its surface glycoprotein hemagglutinin (HA) has been shown to trigger inflammatory programmed cell death in human and murine macrophages in a TLR4- and TNF-dependent manner [98]. Additionally, rhein, an anthraquinone compound derived from traditional herbal medicines, has been demonstrated to possess anti-inflammatory, antioxidant, antitumor, antiviral, and anti-influenza A virus activities, reducing oxidative stress and modulating TLR4, Akt, MAPK, and NF-κB signaling pathways [99]. Moreover, direct activation of TLR4 by IAV HA has been documented, as has indirect activation via the induction of endogenous damage-associated molecular patterns (DAMPs) following IAV infection. For instance, S100A9 has been identified as a molecular danger pattern that activates the TLR4-MyD88 pathway-mediated inflammation during IAV infection [100].

The in silico studies presented here complement the understanding of TLR4 activation by dimerization induced not only by HA2 from influenza A but also for influenza B, as well as for the HA2 analog of influenza C, namely, the hemagglutinin-esterase-fusion (HEF) protein [101].

In the context of various highly pathogenic viruses with established TLR4 activation capabilities (such as Ebola virus and RSV), this study introduces a novel hypothesis that the fusion complex structures of highly pathogenic coronaviruses, including SARS-CoV, MERS-CoV, and SARS-CoV-2, similarly bind to and dimerize TLR4/MD-2 heterocomplexes, leading to the activation of TLR4-triggered signaling pathways such as NF-κB via MyD88 activation and IFN release via TRIF-mediated activation. Although the activation of TLR4 by the SARS-CoV-2 spike protein has been previously documented, it has mostly involved spike protein trimers or individual S1 and S2 subunits. Given the large molecular structure of the spike protein and its components, it can be postulated that TLR4 activation occurs primarily extracellularly at the cell surface. The current study adds a new dimension to this activation, suggesting that intracellular activation pathways of TLR4 within endosomes may also contribute to overall TLR4 activation. Future studies are needed to elucidate the impact of intracellular versus extracellular activation of TLR4 by spike protein components, particularly concerning the levels of TLR4 activation required to induce an effective antiviral immune response versus systemic hyperactivation of TLR4 observed in critical stage patients with SARS-CoV-2 or SARS-CoV infections.

The comparatively slightly lower binding affinity of the SARS-CoV-2 HR1HR2 fusion structure relative to its SARS-CoV and MERS-CoV counterparts leads to speculation that these differences may correlate with the relatively lower incidence of critical stages observed in patients infected with SARS-CoV-2 compared to those infected with SARS-CoV or MERS-CoV. Additionally, a lower ratio of endosomal TLR4 activation by the SARS-CoV-2 spike HR1HR2 complex, in comparison to extracellular TLR4 activation by the SARS-CoV-2 spike protein trimers or subunits, may contribute to a reduced level of TRIF pathway activation—resulting in lower IRF3 and IFN release—compared to the significant release of pro-inflammatory cytokines associated with NF-κB pathway activation (including TNFα, IL-1, IL-6, and IL-8) typically observed during COVID-19 [102,103].

The limitations of this study are primarily related to the fact that the analyses conducted are exclusively in silico. The hypotheses generated from the structural alignment and docking data presented here await experimental validation but may serve as a trigger for further experimental studies aimed at developing strategies to control excessive immune activation.

## 4. Materials and Methods

### 4.1. Data Acquisition and Structural Templates

Amino acid sequences and three-dimensional (3D) macromolecular structures were retrieved from the RCSB Protein Data Bank (PDB; rcsb.org, accessed August–October 2025). The specific entries utilized for receptor and ligand modeling are detailed in Table 5. For the Ebola virus glycoprotein (PDB 5F1B), only chain 2 (GP2) from model 1 was extracted for downstream analysis based on its dimeric configuration, showing a docking score of −284.96, a confidence score of 0.9370, and a ligand root-mean-square deviation (RMSD) of 255.6 Å.

### 4.2. Homology Modeling

Sequence similarity searches against the PDB database were performed using the HH-suite package to identify homologous templates for receptors and ligands. Homology models were generated using MODELLER (https://salilab.org, accessed 12 October 2025), with sequence alignments executed via ClustalW (https://ebi.ac.uk, accessed 12 October 2025). The crystal structure of the human TLR4 polymorphic variant (D299G and T399I) complexed with MD-2 and LPS (PDB 4G8A) served as the receptor template [104]. The post-fusion hairpin conformation of the SARS-CoV spike glycoprotein (PDB 1WYY) was utilized as the ligand template [105].

### 4.3. In Silico Protein–Protein Docking

Molecular docking simulations were performed using the HDOCK server (http://hdock.phys.hust.edu.cn), which integrates template-based and template-free hybrid protocols [60,106,107,108]. Global docking sampling was executed via HDOCKlite, a fast Fourier transform (FFT)-based hierarchical program utilizing an optimized shape-based pairwise scoring function. During sampling, the ligand grid potential accounted for contributions from neighboring receptor grids using an exponential decay function (e-1/r^2^), where r represents the distance between the ligand and receptor grids. Rotational sampling was performed at a 15° angle interval, coupled with a 1.2 Å translational grid spacing for the FFT-based search. For each rotation, the top 10 translations demonstrating optimal shape complementarity were refined using knowledge-based scoring functions [60]. Each docking run generated 4392 evenly distributed orientations, representing the total sampled binding modes.

### 4.4. Clustering and Scoring Criteria

The generated binding modes were clustered using a backbone-atom root-mean-square deviation (RMSD) cutoff of 5 Å. For pairs scoring below this threshold, only the pose with the superior energy score was retained [108]. Binding affinity was evaluated using the internally calculated HDOCK docking score and an empirical confidence score. A docking score of less than −200 indicates potential binding, with increasingly negative values correlating with a higher thermodynamic probability of interaction. A confidence score of >0.7 indicates a high likelihood of interaction. All final structural models selected for analysis exhibited a docking score of less than −200 and a confidence score > 0.8. Structural interface analysis was completed by extracting all receptor–ligand residue pairs positioned within a 5.0 Å cutoff radius.

### 4.5. Structural Alignment and Visualization

Macromolecular structural alignments were performed directly within the RCSB PDB platform. Furthermore, molecular visualizations, structural figures, and spatial analyses were generated using the Mol* (MolStar) viewer v5.0.0 (https://molstar.org/viewer/, 12 October 2025) [109].

## 5. Conclusions

The in silico analyses presented herein provide a novel hypothesis for TLR4 activation by SARS-CoV-2. Our structural data suggest that the SARS-CoV-2 HR1HR2 fusion core complex can bind to the TLR4/MD-2 complex through specific salt bridges, alongside multiple polar and non-polar interactions involving both dimerization partners. This interaction potentially facilitates the dimerization of two TLR4/MD-2 complexes. Notably, this dimerization potential was impaired in the Omicron variant of the SARS-CoV-2 HR1HR2 fusion core complex compared to the wild-type virus.

Furthermore, the SARS-CoV-2 HR1HR2 fusion core complex exhibits high structural similarity to class I fusion complexes from other highly pathogenic viruses, including SARS-CoV, MERS-CoV, influenza viruses A, B, and C, respiratory syncytial virus (RSV), and partially Ebola virus. Importantly, the capability to induce TLR4/MD-2 dimerization via their respective fusion complexes was suggested by the present docking studies to be a shared feature among various highly pathogenic viruses. While these structural alignment and molecular docking findings require experimental validation, they may offer a valuable framework for understanding viral pathogenesis. These results may serve as a starting point for future experimental approaches to elucidate the precise molecular interactions driving TLR4/MD-2 recognition during viral infection. Unraveling these mechanisms is essential to understanding host innate immune recognition, as well as the pathways that trigger detrimental immune hyperactivation.

Importantly, these findings highlight the therapeutic potential of TLR4 inhibitors. Target-specific downmodulation of TLR4 signaling—either through small-molecule antagonists, peptide mimetics, or neutralizing antibodies—could disrupt the stabilizing network of salt bridges, polar and hydrophobic interactions and prevent receptor dimerization. Such interventions offer a promising strategy to mitigate the devastating hyperinflammation and cytokine storms associated with severe viral infections without entirely abolishing baseline host defense mechanisms.

## Figures and Tables

**Table 1:** Thermodynamic scoring parameters, confidence scores, and root-mean-square deviations (RMSD) of the top 10 ranked in silico docking models representing the interaction between the SARS-CoV-2 HR1HR2 fusion core complex (PDB 8CZI) and the human TLR4/MD-2 heterodimer (PDB 3FXI).

Summary of the Top 10 Models										
Rank	1	2	3	4	5	6	7	8	9	10
Docking score	−252.41	−243.90	−233.53	−227.24	−225.90	−224.04	−222.54	−221.51	−219.59	−219.01
Confidence Score	0.8858	0.8674	0.8417	0.8242	0.8202	0.8147	0.8101	0.8069	0.8009	0.7990
Ligand rmsd (Å)	244.09	143.49	144.94	146.89	244.52	170.62	194.07	214.34	187.32	158.72
Interface residues	Model 1	Model 2	Model 3	Model 4	Model 5	Model 6	Model 7	Model 8	Model 9	Model 10

**Table 2:** Comparative in silico TLR4/MD-2 homodimerization frequencies across analyzed ancestral and mutated SARS-CoV-2 HR1HR2 fusion core lineages.

Lineage/Variant	PDB ID	Key Substitutions in Fusion Core	Dimerization Frequency (Top 10 Models)
Wild-Type (Ancestral)	8CZI/7RZQ	None (Baseline)	4/10
D936Y Variant	7RZR	D936Y	2/10
Q954H Intermediate	8VYA	Q954H	4/10
N969K Single Mutant	8FA1	N969K	1/10
Omicron Lineage	7TIK	Q954H, N969K, L981F	0/10

**Table 3:** Docking of different Coronavirus fusion complexes to human TLR4/MD-2.

Virus PDB Nr.	Structure	Binding to hTLR4 HDOCK (First 10 Models Only)	Docking Score (Model)
SARS-CoV-2 8CZI	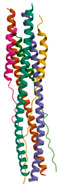	SARS-CoV-2 HR1HR2 fusion core complex 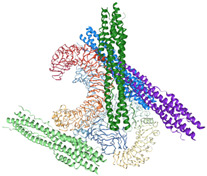	−224 (6) −223 (7) −222 (8) −220 (9)
SARS-CoV-1 5ZVM	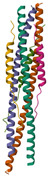	Human Coronavirus SARS HR1 motif 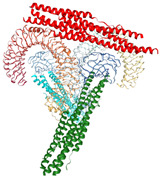	−241 (3) −229 (5) −217 (7)
MERS-CoV 5ZVK	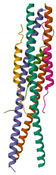	Human Coronavirus MERS HR1 motif 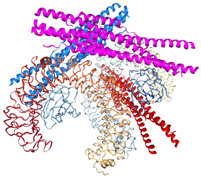	−238 (3) −226 (5) −225 (6)

**Table 4:** Docking of different viral fusion complexes to TLR4/MD-2.

Virus PDB Nr.	Structure	Binding to hTLR4 HDOCK (First 10 Models Only)	Docking Score (Model)
RSV 6OJ7	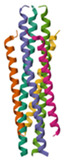	RSV fusion glycoprotein N-terminal heptad repeat domain 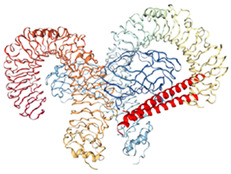	−253 (1) −239 (3) −236 (5)
Influenza 4OXM	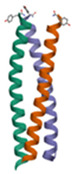	Central Coiled-Coil from Influenza Hemagglutinin HA2 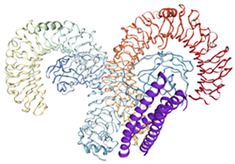	−230 (6) −226 (8) −223 (10)
Influenza B 4NKJ	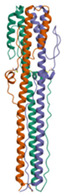	Influenza B virus hemagglutinin at membrane fusion pH 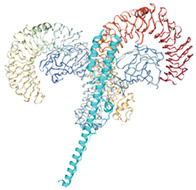	−252 (5) −244 (9)
Influenza C 6WKO	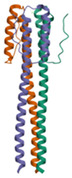	Influenza C virus hemagglutinin-esterase-fusion (HEF2) intermediate 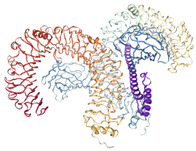	−252 (5) −245 (6) −243 (8) −243 (9) −239 (10)
Ebola virus 5F1B_2	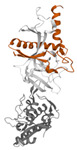	Ebola virus glycoprotein (GP1 grey & GP2 (red)) bound to its endosomal receptor Niemann-Pick C1 (blue) 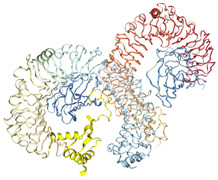	−283 (1)

**Table 5:** Protein Data Bank (PDB) entries utilized for structural modeling and docking studies.

PDB ID	Description/Macromolecular Complex	Reference
3FXI	Human TLR4–human MD-2 complex	[59]
8CZI	SARS-CoV-2 HR1HR2 fusion core complex (extended HR2)	[58]
7RZQ	SARS-CoV-2 HR1HR2 fusion core complex	[61]
2BEZ	Proteolytically resistant core, SARS-CoV S2 fusion protein	[62]
6M1V	Post-fusion core, 2019-nCoV S2 subunit	[63]
7RZR	SARS-CoV-2 HR1HR2 fusion core complex with D936Y mutation	[61]
8VYA	SARS-CoV-2 Omicron Variant Spike Glycoprotein Fusion Core (Q954H)	[64]
7TIK	Structure of the SARS-CoV-2 Omicron spike post-fusion bundle	[65]
8FA1	SARS-CoV-2 HR1HR2 fusion core complex with N969K mutation	[65]
5ZVM	Human coronavirus SARS HR1 motif bound to pan-CoVs inhibitor EK1	[66]
5ZVK	MERS-CoV HR1 motif bound to pan-CoVs inhibitor EK1	[66]
6OJ7	RSV fusion glycoprotein N-terminal heptad repeat domain (VIQKI I456F)	[67]
4OXM	Central coiled-coil from Influenza Hemagglutinin HA2	[68]
4NKJ	Influenza B virus hemagglutinin at membrane fusion pH	[69]
6WKO	Influenza C virus hemagglutinin-esterase-fusion (HEF2) intermediate	[70]
5F1B	Ebola virus glycoprotein bound to endosomal receptor NPC1	[71]

## Data Availability

The original contributions presented in this study are included in the article/Supplementary Materials. Further inquiries can be directed to the author.

## Supplementary Materials

The following supporting information can be downloaded at: https://www.mdpi.com/article/10.3390/ijms27125317/s1.
