# Supplementary material for: In Silico Modeling of Structural Compatibility and Alignment Between Viral Class I Fusion Cores and Human TLR4/MD-2
Source: Int J Mol Sci. 2026 Jun 12;27(12):5317. doi: 10.3390/ijms27125317 (PMC13299227; doi:10.3390/ijms27125317)
Supplement: Supplementary file 1 [file ijms-27-05317-s001.zip › ijms-4233080-supplementary.pdf]

Supplementary data

Suppl Fig S1: **7RZQ** Cryo-EM structure of the SARS-CoV-2 HR1HR2 fusion core

complex PDB DOI: https://doi.org/10.2210/pdb7RZQ/pdb

Table S-I

Summary of the Top 10 Models

Rank	1	2	3	4	5	6	7	8	9	10
Docking Score	-257.90	-253.97	-239.18	-232.57	-228.75	-219.18	-217.02	-215.04	-214.80	-213.39
Confidence Score	0.8964	0.8889	0.8561	0.8391	0.8285	0.7996	0.7926	0.7860	0.7852	0.7804
Ligand rmsd (Å)	193.47	142.63	160.34	193.37	242.12	222.78	191.14	223.16	159.67	136.59
Interface residues	model_1	model_2	model_3	model_4	model_5	model_6	model_7	model_8	model_9	model_10

Fig. S-1

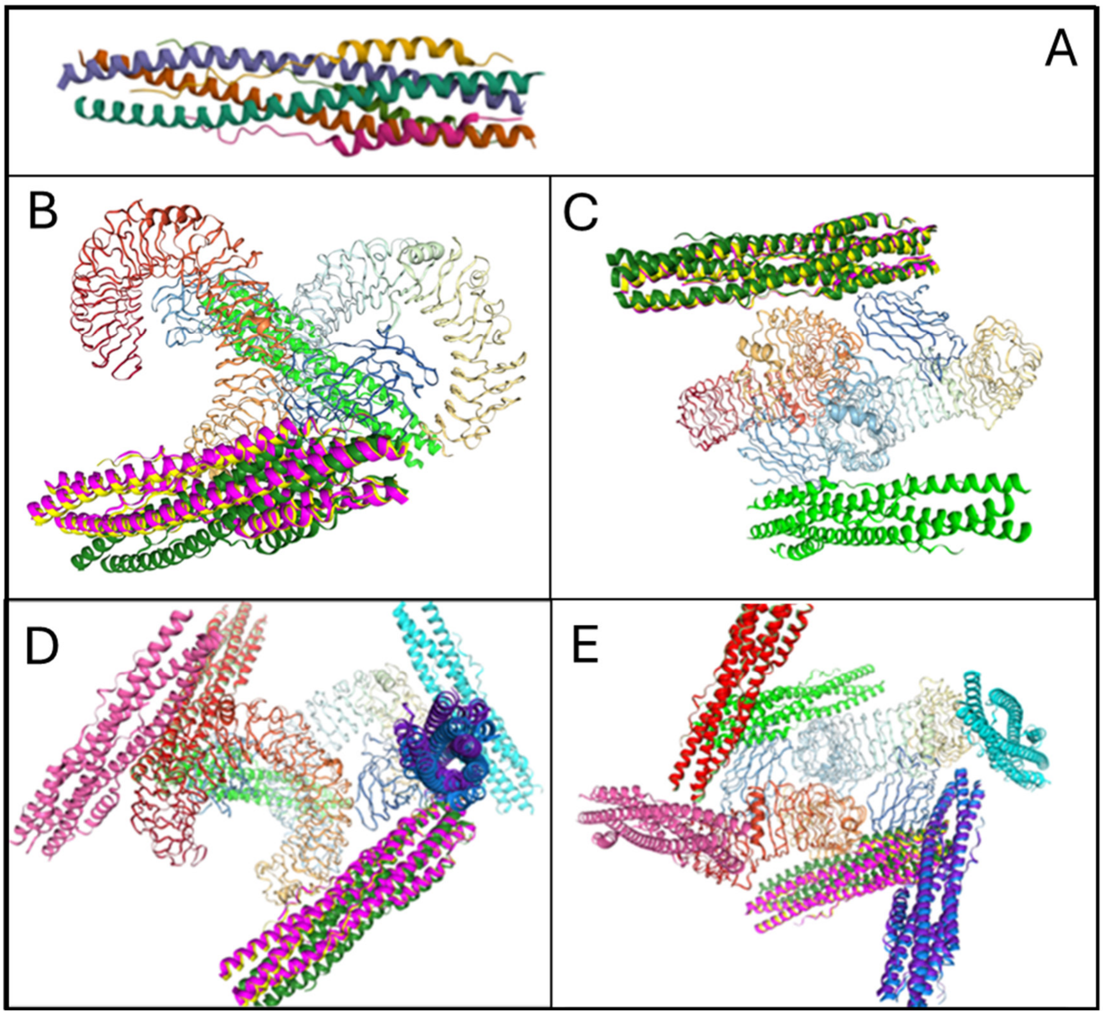

Legend Fig. S-1:

Docking studies (HDOCK) were performed using PDB-derived SARS-CoV-2 HR1HR2 fusion core structure model **7RZQ** (Cryo-EM structure of the SARS-CoV-2 HR1HR2 fusion core complex (**A**) [**61**] PDB DOI: https://doi.org/10.2210/pdb7RZQ/pdb) with **3FXI** (Crystal structure of the human TLR4-human MD-2, PDB DOI: https://doi.org/10.2210/pdb3FXI/pdb) [**59**].

Four out of the top ten prediction models (models 1,2,4,7) were found to confer dimerization of the TLR4/MD-2 heterodimers (**B**) side view, (**C**) top view), and 6 models (3,5,6,8,9,10) were found to bind only to TLR4 or only to MD-2, respectively, not leading to dimerization of the TLR4/MD-2 heterocomplex, all 10 models depicted together in (**D**) side view and (**E**) top view.

Suppl Fig S2: **7RZR** Cryo-EM structure of the SARS-CoV-2 HR1HR2 fusion core complex with D936Y mutation

PDB DOI: https://doi.org/10.2210/pdb7RZR/pdb

Table S-II

Summary of the Top 10 Models

Rank	1	2	3	4	5	6	7	8	9	10
Docking Score	-244.04	-242.99	-242.31	-236.10	-235.05	-234.61	-230.00	-228.88	-228.37	-228.19
Confidence Score	0.8677	0.8653	0.8637	0.8484	0.8457	0.8445	0.8320	0.8289	0.8274	0.8269
Ligand rmsd (Å)	233.17	147.35	146.84	206.27	231.36	164.28	154.70	233.87	191.84	135.77
Interface residues	model_1	model_2	model_3	model_4	model_5	model_6	model_7	model_8	model_9	model_10

Fig. S-2

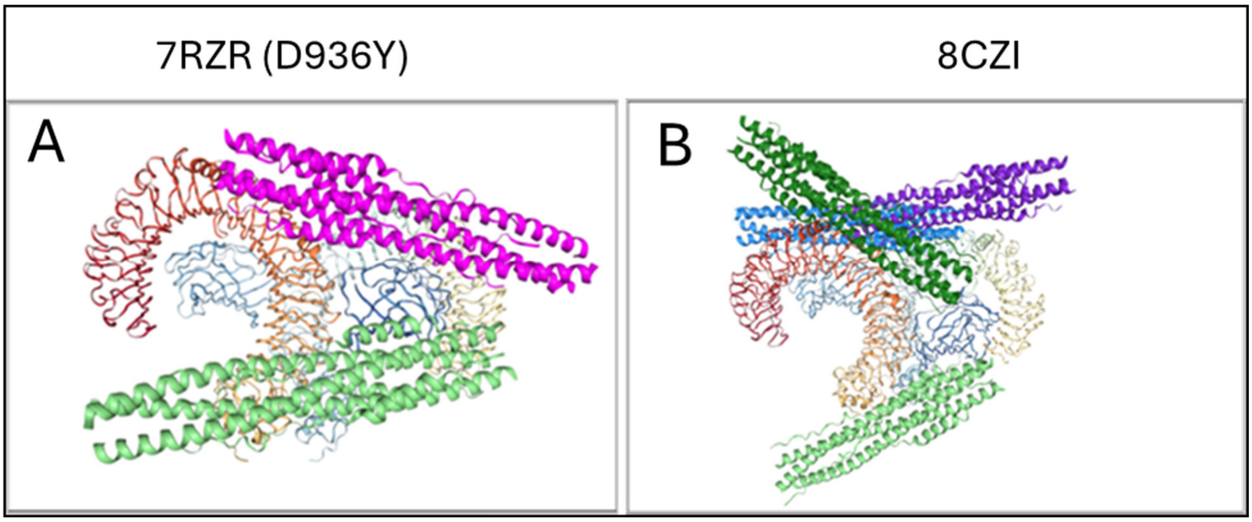

Legend Fig. S-2:

Docking studies were performed using PDB-derived SARS-CoV-2 HR1HR2 fusion core structure model **7RZQ** (Cryo-EM structure of the SARS-CoV-2 HR1HR2 fusion core complex [**61**] or with **7RZR** (Cryo-EM structure of the SARS-CoV-2 HR1HR2 fusion core complex with D936Y mutation, PDB DOI: https://doi.org/10.2210/pdb7RZR/pdb) [**64**] with **3FXI** (Crystal structure of the human TLR4-human MD-2 (without LPS ligand), PDB DOI: https://doi.org/10.2210/pdb3FXI/pdb) [**59**].

The effect of the single D936Y substitution on dimerization potency for TLR4-MD2 complex can be seen. The dimerization potency was significantly lower for SARS-CoV-2 HR1HR2 fusion complex with D936Y mutation with only 2/10 models (models: 4,9) showing dimerization (**A**) compared to the wild-type SARS-CoV-2 HR1HR2 fusion core complex (8CZI) with 4/10 of the models fitting with dimerization (**B**).

Suppl Fig S3: **8VYA** SARS-CoV-2 Omicron Variant Spike Glycoprotein Fusion Core (Q954H) PDB DOI: https://doi.org/10.2210/pdb8VYA/pdb

Table S-III

Summary of the Top 10 Models

Rank	1	2	3	4	5	6	7	8	9	10
Docking Score	-254.04	-239.70	-236.65	-235.72	-235.59	-235.26	-233.44	-231.22	-228.28	-227.44
Confidence Score	0.8890	0.8574	0.8498	0.8474	0.8471	0.8462	0.8414	0.8354	0.8272	0.8247
Ligand rmsd (Å)	56.29	89.96	108.41	72.32	52.52	46.38	99.44	81.34	52.65	87.03
Interface residues	model_1	model_2	model_3	model_4	model_5	model_6	model_7	model_8	model_9	model_10

Fig. S-3

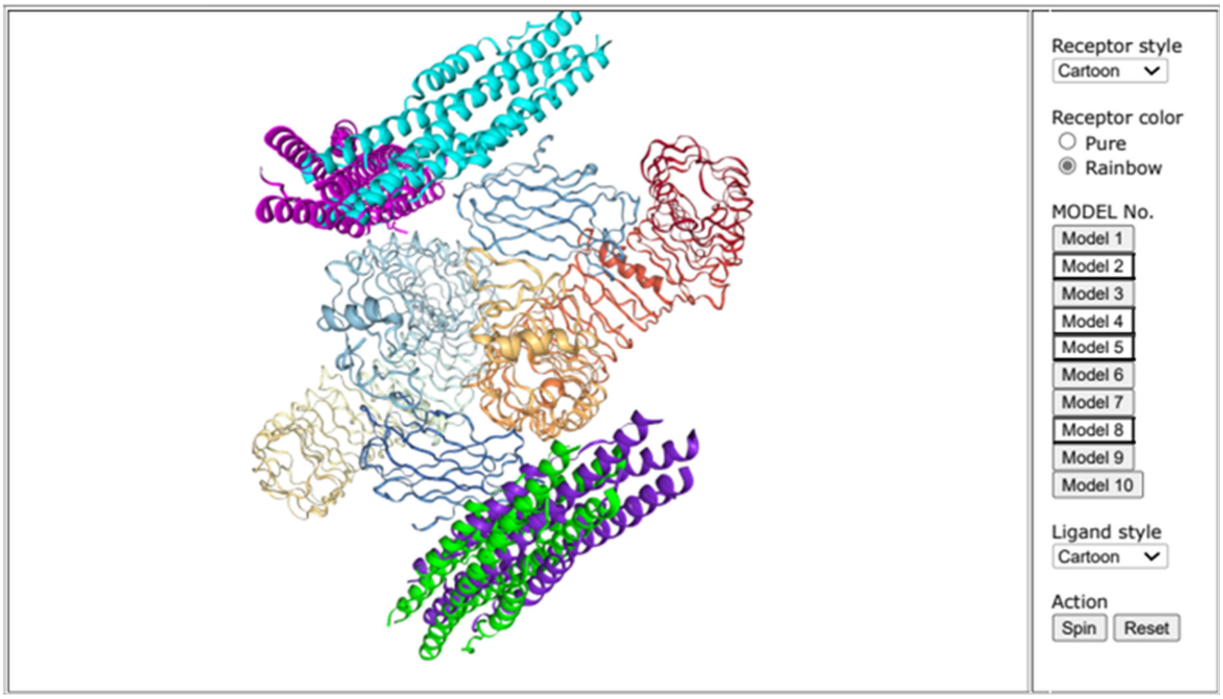

Legend Fig. S-3:

Docking studies were performed using PDB-derived SARS-CoV-2 HR1HR2 fusion core structure model **8VYA (**SARS-CoV-2 Omicron Variant Spike Glycoprotein Fusion Core (Q954H), PDB DOI: https://doi.org/10.2210/pdb8VYA/pdb [**64**]) with one Omicron-typical amino acid substitution, i.e. Q954H [**65**] with **3FXI** (Crystal structure of the human TLR4-human MD-2 (without LPS ligand), PDB DOI: https://doi.org/10.2210/pdb3FXI/pdb) [**59**].

There was no effect of this single Q954H substitution on dimerization potency for TLR4-MD2 compared to the wild-type SARS-CoV-2 HR1HR2 fusion core complex (**8CZI, see Fig. 1C**) with 4/10 of the top 10 models fitting with TLR4/MD-2 dimerization.

Suppl Fig S4: **7TIK** Structure of the SARS-CoV-2 Omicron spike post-fusion

bundle PDB DOI: https://doi.org/10.2210/pdb7TIK/pdb

Table S-IV

Summary of the Top 10 Models

Rank	1	2	3	4	5	6	7	8	9	10
Docking Score	-297.67	-297.06	-293.55	-286.73	-266.77	-264.89	-255.78	-251.61	-239.11	-238.81
Confidence Score	0.9504	0.9498	0.9464	0.9390	0.9118	0.9087	0.8924	0.8841	0.8560	0.8552
Ligand rmsd (Å)	243.24	145.68	243.65	144.83	244.36	145.73	243.55	146.13	225.63	137.97
Interface residues	model 1	model 2	model 3	model 4	model 5	model 6	model 7	model 8	model 9	model 10

Fig. S-4

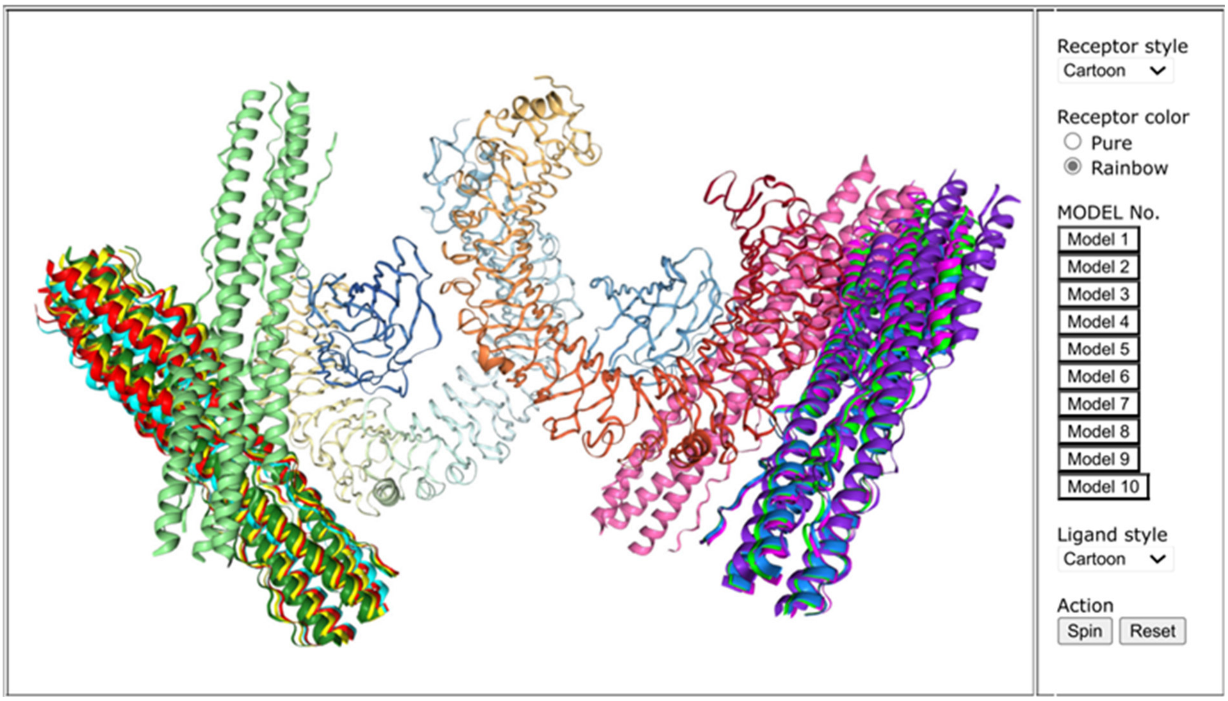

Legend Fig. S-4:

Docking studies were performed using PDB-derived SARS-CoV-2 Omicron spike HR1HR2 fusion core structure model **7TIK (**Structure of the SARS-CoV-2 Omicron spike post-fusion bundle, PDB DOI: https://doi.org/10.2210/pdb7TIK/pdb]) with three S2 Omicron-typical amino acid substitutions, i.e. Q954H, N969K, and L981F [**66**] with **3FXI** (Crystal structure of the human TLR4-human MD-2 (without LPS ligand), PDB DOI: https://doi.org/10.2210/pdb3FXI/pdb) [**59**].

The dimerization potency of the Omicron S2 derived post-fusion bundle was dramatically affected, with no dimerization potency for TLR4-MD2 found among the top 10 binding models (0/10) compared to the wild-type SARS-CoV-2 HR1HR2 fusion core complex (4/10 of the top 10 models fitting with TLR4/MD-2 dimerization (**8CZI, see Fig. 1C**)).

Suppl Fig S5: **8FA1** Cryo-EM structure of the SARS-CoV-2 HR1HR2 fusion core complex with N969K mutation

PDB DOI: https://doi.org/10.2210/pdb8FA1/pdb

Table S-V

Summary of the Top 10 Models

Rank	1	2	3	4	5	6	7	8	9	10
Docking Score	-239.94	-235.33	-231.59	-228.23	-227.85	-227.53	-225.76	-223.92	-223.65	-223.29
Confidence Score	0.8580	0.8464	0.8364	0.8270	0.8259	0.8250	0.8198	0.8143	0.8135	0.8124
Ligand rmsd (Å)	156.24	156.45	237.78	168.72	137.73	120.69	148.80	174.18	195.87	135.25
Interface residues	model 1	model 2	model 3	model 4	model 5	model 6	model 7	model 8	model 9	model 10

Fig. S-5

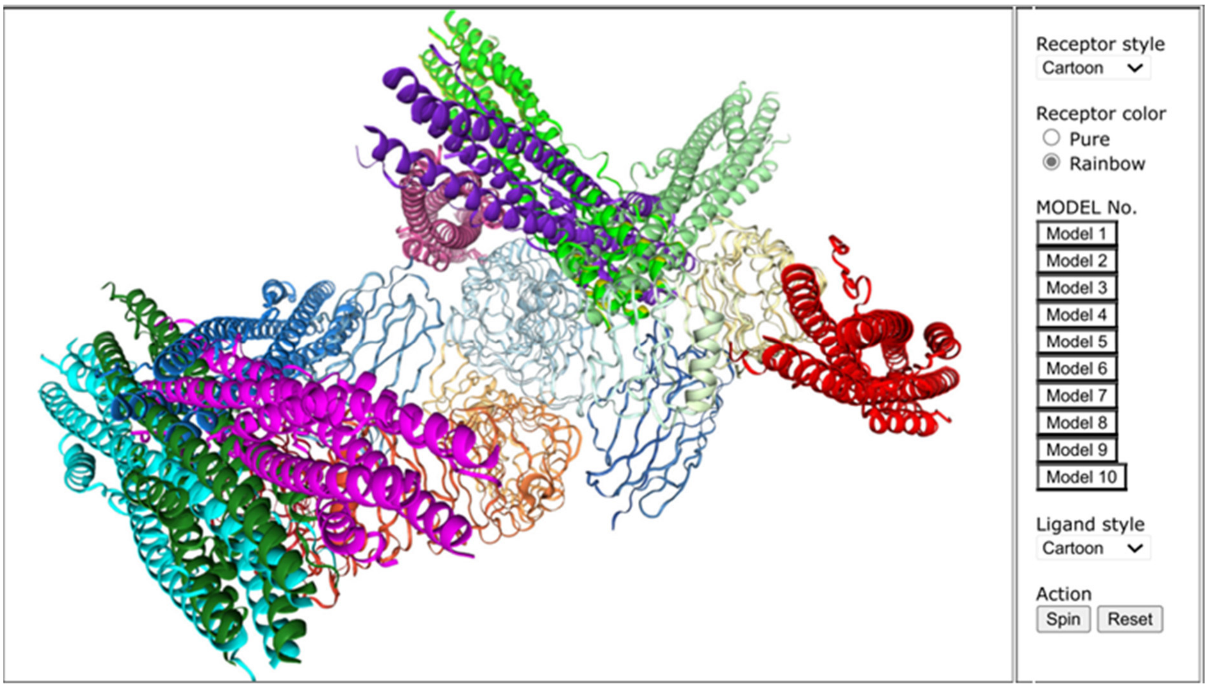

Legend Fig. S-5:

Docking studies were performed using PDB-derived SARS-CoV-2 Omicron spike HR1HR2 fusion core structure model **8FA1 (**Cryo-EM structure of the SARS-CoV-2 HR1HR2 fusion core complex with N969K mutation PDB DOI: https://doi.org/10.2210/pdb8FA1/pdb) with one Omicron-typical amino acid substitution, i.e. N969K [**67**] with **3FXI** (Crystal structure of the human TLR4-human MD-2 (without LPS ligand), PDB DOI: https://doi.org/10.2210/pdb3FXI/pdb) [**59**].

The dimerization potency of the Omicron S2 derived post-fusion bundle was significantly affected, with only one dimerization potency for TLR4-MD2 found among the top 10 binding models (1/10) compared to the wild-type SARS-CoV-2 HR1HR2 fusion core complex (4/10 of the top 10 models fitting with TLR4/MD-2 dimerization (**8CZI, see Fig. 1C**)).

Suppl. Fig. S-6

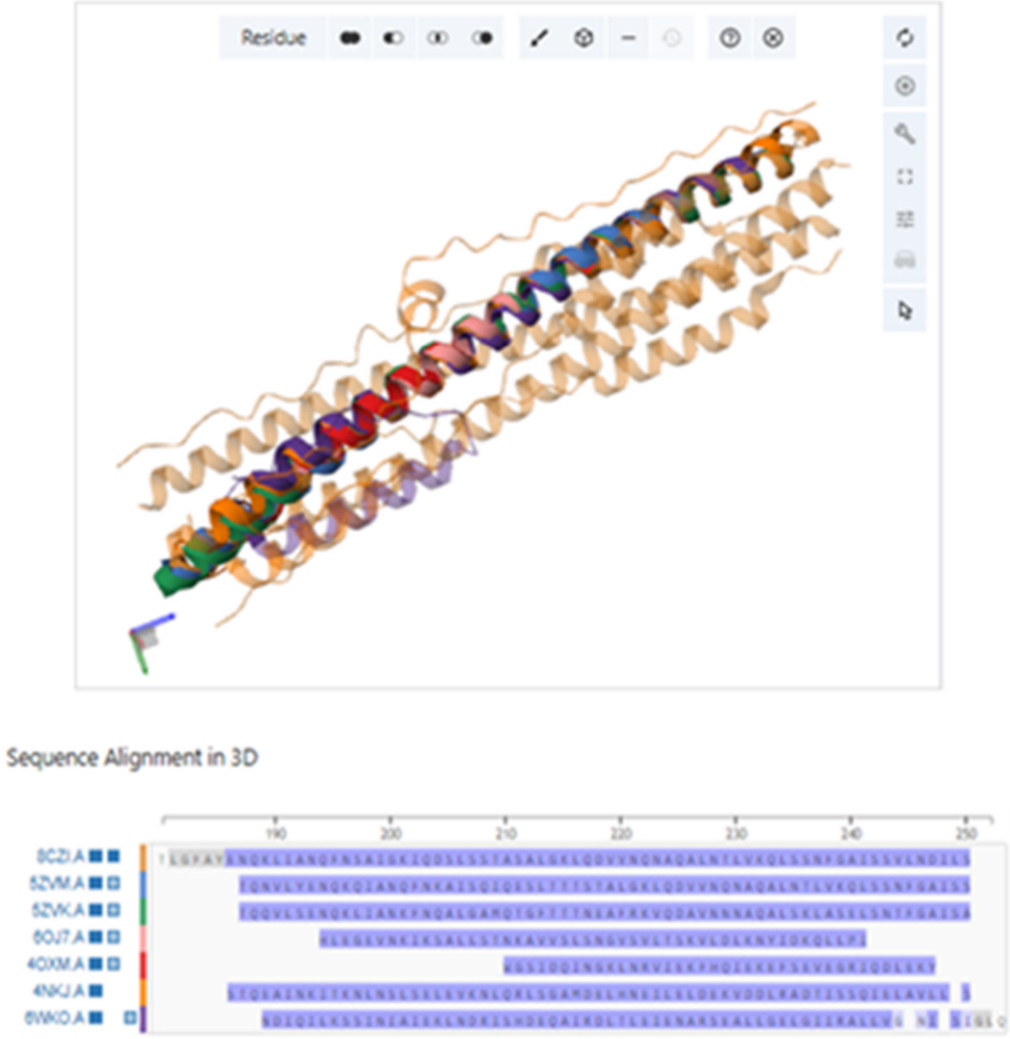

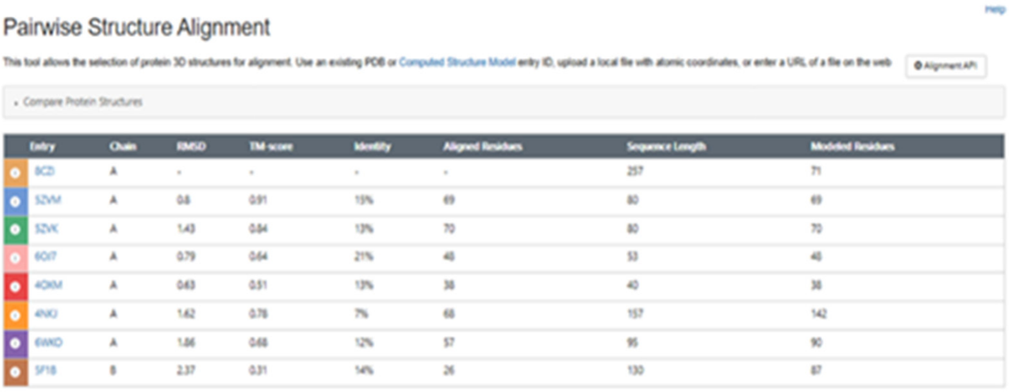

Pairwise Structure Alignment

This tool allows the selection of protein 3D structures for alignment. Use an existing PDB or Computed Structure Model entry ID, upload a local file with atomic coordinates, or enter a URL of a file on the web

Entry	Chain	RMSD	TM-score	Identity	Aligned Residues	Sequence Length	Modeled Residues
8CZI	A	-	-	-	-	257	71
5ZVM	A	0.8	0.91	15%	69	80	69
5ZVK	A	1.43	0.84	13%	70	80	70
6OJ7	A	0.79	0.64	21%	48	53	48
4OXM	A	0.63	0.51	13%	38	40	38
4NKJ	A	1.62	0.78	7%	68	157	142
6WKO	A	1.86	0.68	12%	57	95	90
5F18	B	2.37	0.31	14%	26	130	87

Fig. S-6 Pairwise alignment of various viral fusion proteins to SARS-CoV-2 spike protein HR1HR2:

Pairwise alignment of SARS-CoV-2 spike protein HR1HR2 fusion core complex (8CZI) with Human Coronavirus SARS-CoV spike protein HR1 motif (5ZVM), Human Coronavirus MERS-CoV spike protein HR1 (5ZVK), Respiratory syncytial virus fusion glycoprotein N-terminal heptad repeat domain (6OJ7), Coiled-Coil from Influenza Hemagglutinin HA2 (4OXM), influenza B virus hemagglutinin at membrane fusion pH (4NKJ), influenza C virus hemagglutinin-esterase-fusion (HEF2) intermediate (6WKO); all depicted as monomers (except 8CZI as trimer).

Suppl. Fig. S-7

A: Docking 3FXI with 5ZVM (Crystal Structure of the Human Coronavirus SARS HR1)

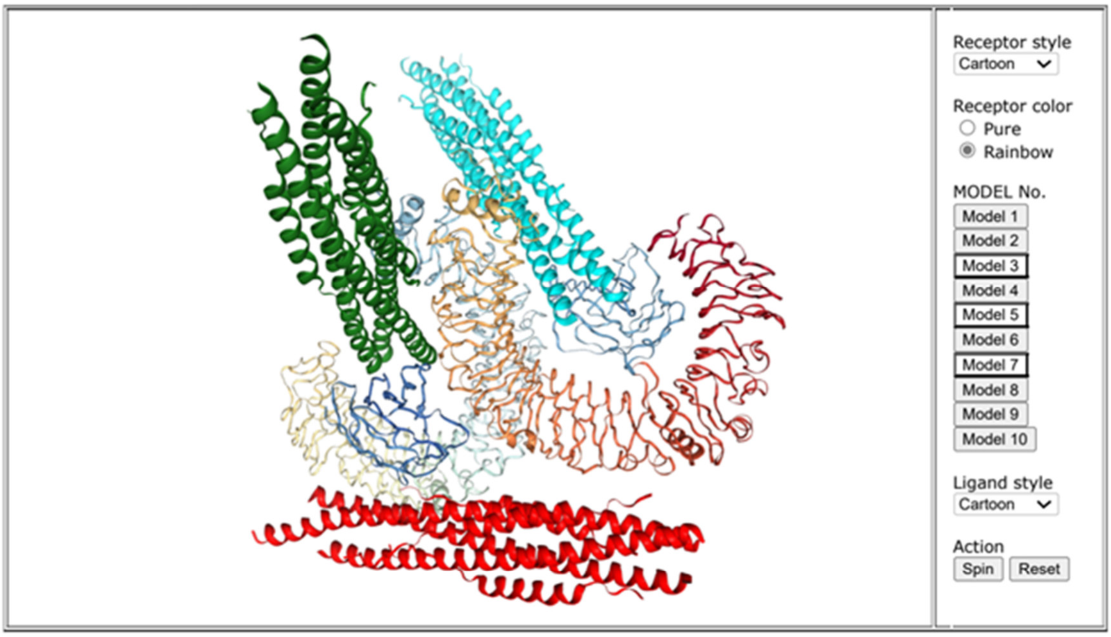

Complex Template Information (Click to Show)

Summary of the Top 10 Models

Rank	1	2	3	4	5	6	7	8	9	10
Docking Score	-248.69	-243.07	-240.85	-232.22	-229.21	-217.37	-217.02	-214.52	-214.34	-213.88
Confidence Score	0.8780	0.8655	0.8602	0.8381	0.8298	0.7937	0.7926	0.7842	0.7836	0.7820
Ligand rmsd (Å)	374.16	307.09	357.70	317.32	303.70	370.13	369.64	373.53	319.13	307.20
Interface residues	model 1	model 2	model 3	model 4	model 5	model 6	model 7	model 8	model 9	model 10

B: Docking 3FXI with 5ZVK (Crystal Structure of the Human Coronavirus MERS HR1 motif)

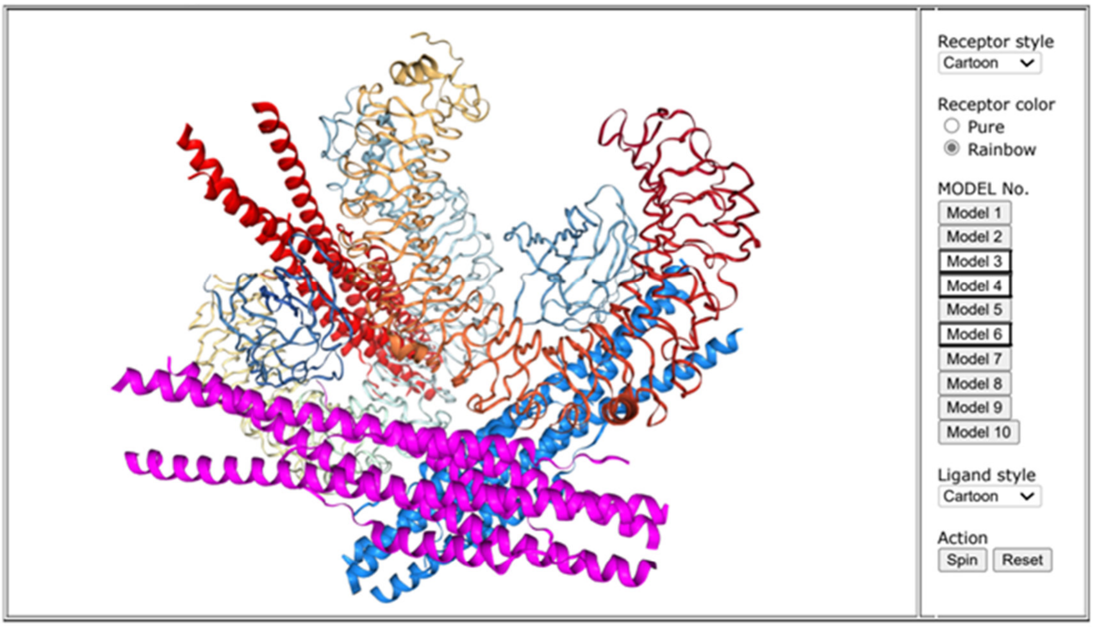

Complex Template Information (Click to Show)

Summary of the Top 10 Models

Rank	1	2	3	4	5	6	7	8	9	10
Docking Score	-257.32	-254.95	-238.42	-226.03	-225.83	-225.30	-220.48	-220.31	-218.80	-218.80
Confidence Score	0.8953	0.8908	0.8543	0.8206	0.8200	0.8185	0.8037	0.8032	0.7983	0.7983
Ligand rmsd (Å)	148.56	151.73	239.19	209.09	151.83	204.38	158.63	146.75	257.64	157.31
Interface residues	model 1	model 2	model 3	model 4	model 5	model 6	model 7	model 8	model 9	model 10

C: Docking 3FXI with 6OJ7 (RSV fusion glycoprotein N-terminal heptad repeat) domain

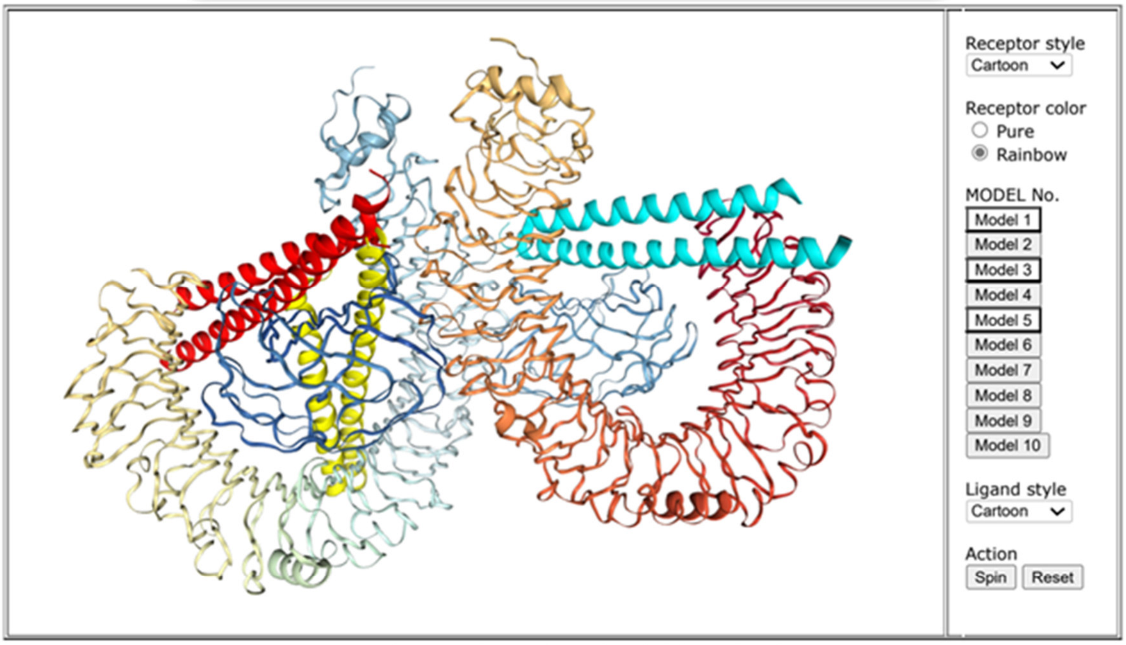

Complex Template Information (Click to Show)

Summary of the Top 10 Models

Rank	1	2	3	4	5	6	7	8	9	10
**Docking Score**	-252.63	-250.70	-239.38	-237.12	-235.64	-234.69	-232.73	-232.30	-231.53	-230.32
**Confidence Score**	0.8862	0.8823	0.8566	0.8510	0.8472	0.8447	0.8395	0.8383	0.8363	0.8329
**Ligand rmsd (Å)**	57.28	72.96	57.09	76.16	39.09	71.70	71.44	55.34	50.95	54.15
**Interface residues**	model_1	model_2	model_3	model_4	model_5	model_6	model_7	model_8	model_9	model_10

E: Docking 3FXI with 6WKO (Influenza C virus hemagglutinin-esterase-fusion (HEF2))

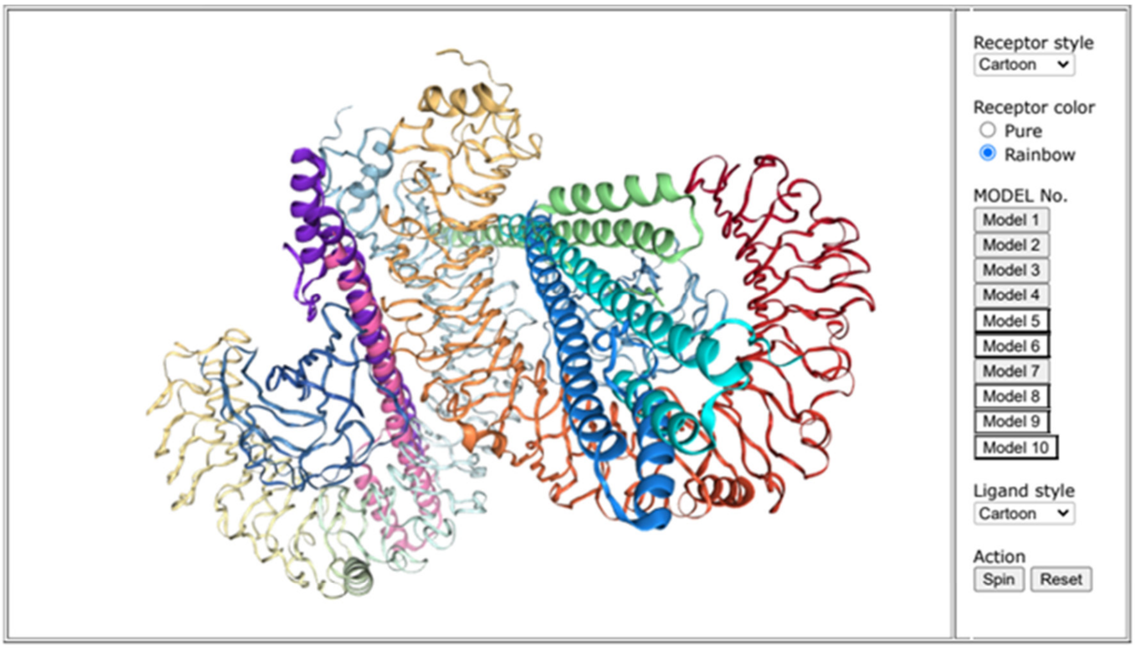

Complex Template Information (Click to Show)

Summary of the Top 10 Models

Rank	1	2	3	4	5	6	7	8	9	10
**Docking Score**	-265.23	-264.81	-260.30	-255.01	-252.33	-244.69	-244.15	-243.15	-242.75	-238.63
**Confidence Score**	0.9093	0.9086	0.9008	0.8909	0.8856	0.8692	0.8680	0.8656	0.8647	0.8548
**Ligand rmsd (Å)**	83.11	78.09	86.34	83.51	58.43	57.13	40.78	57.09	60.14	74.73
**Interface residues**	model_1	model_2	model_3	model_4	model_5	model_6	model_7	model_8	model_9	model_10

F: Docking 3FXI with 4NKJ (Influenza B virus hemagglutinin at membrane fusion pH)

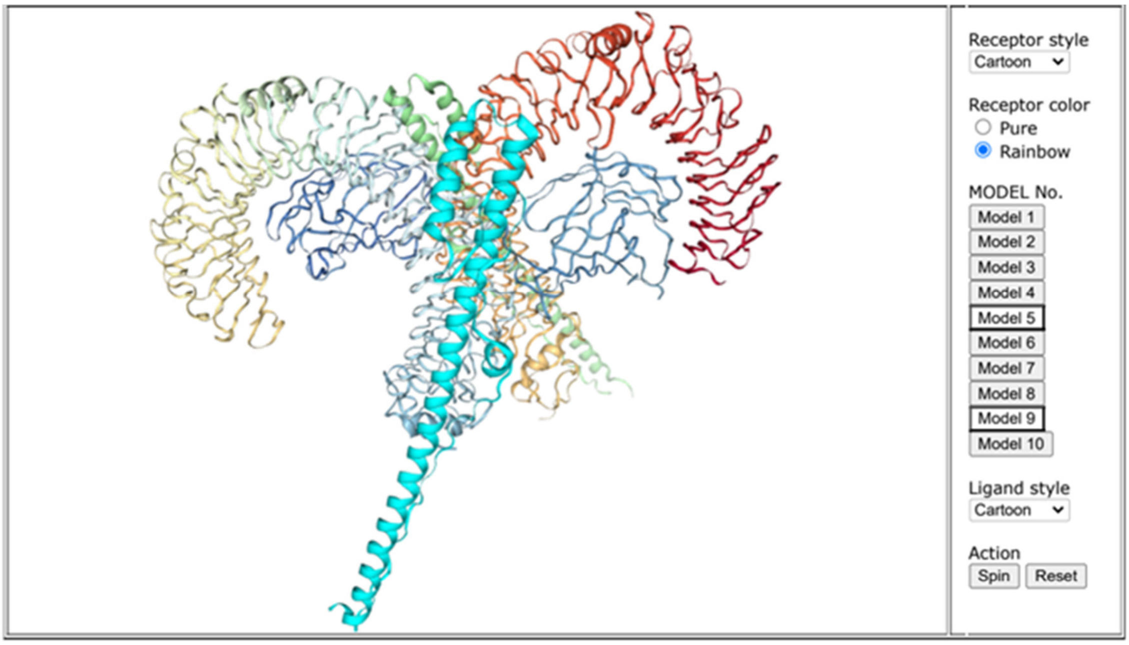

Complex Template Information (Click to Show)

Summary of the Top 10 Models

**Rank**	**1**	**2**	**3**	**4**	**5**	**6**	**7**	**8**	**9**	**10**
**Docking Score**	-275.66	-274.78	-254.07	-253.43	-251.26	-250.38	-248.56	-248.06	-244.33	-243.92
**Confidence Score**	0.9251	0.9238	0.8891	0.8878	0.8834	0.8816	0.8777	0.8767	0.8684	0.8674
**Ligand rmsd (Å)**	90.39	71.74	90.93	95.01	94.62	71.11	72.68	53.20	44.92	105.91
**Interface residues**	model_1	model_2	model_3	model_4	model_5	model_6	model_7	model_8	model_9	model_10

G: Docking 3FXI with 4OMX (Central Coiled-Coil from Influenza Hemagglutinin HA2)

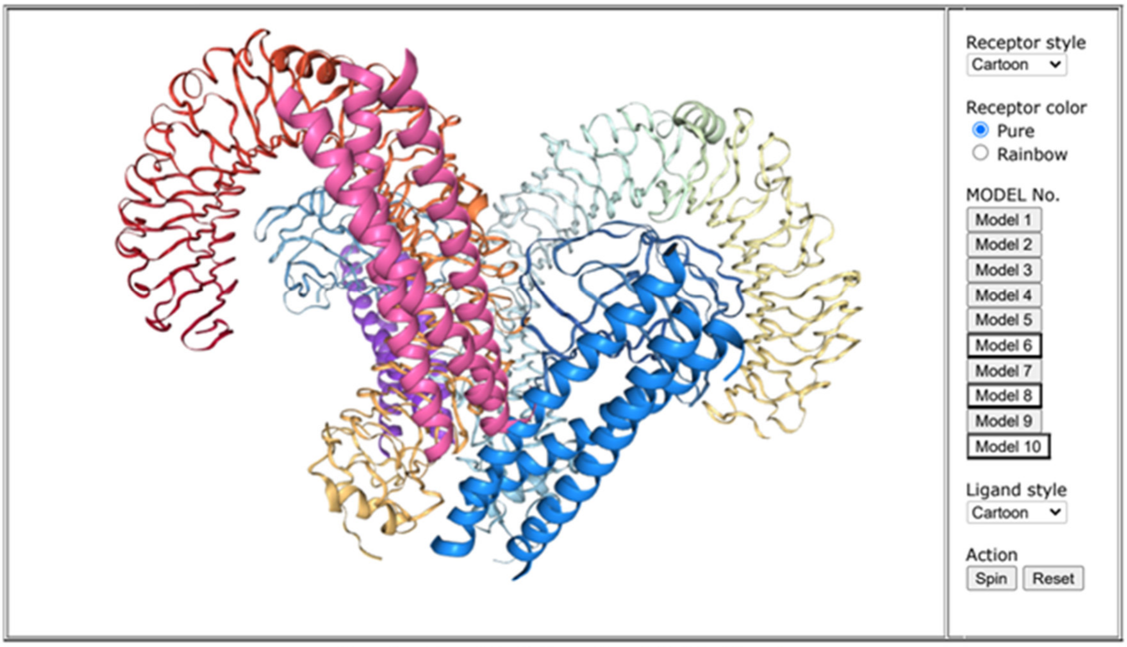

Complex Template Information (Click to Show)

Summary of the Top 10 Models

**Rank**	**1**	**2**	**3**	**4**	**5**	**6**	**7**	**8**	**9**	**10**
**Docking Score**	-258.98	-255.40	-237.91	-233.27	-233.01	-230.24	-228.31	-225.88	-223.41	-223.29
**Confidence Score**	0.8984	0.8917	0.8530	0.8410	0.8403	0.8327	0.8272	0.8202	0.8128	0.8124
**Ligand rmsd (Å)**	41.60	67.46	80.04	58.20	41.62	51.19	102.28	52.14	81.45	61.49
**Interface residues**	model_1	model_2	model_3	model_4	model_5	model_6	model_7	model_8	model_9	model_10

H: Docking 3FXI with 5F1B_2 (Ebola virus GP2) from 5F1B (Ebola virus glycoprotein bound to its endosomal receptor Niemann-Pick C1)

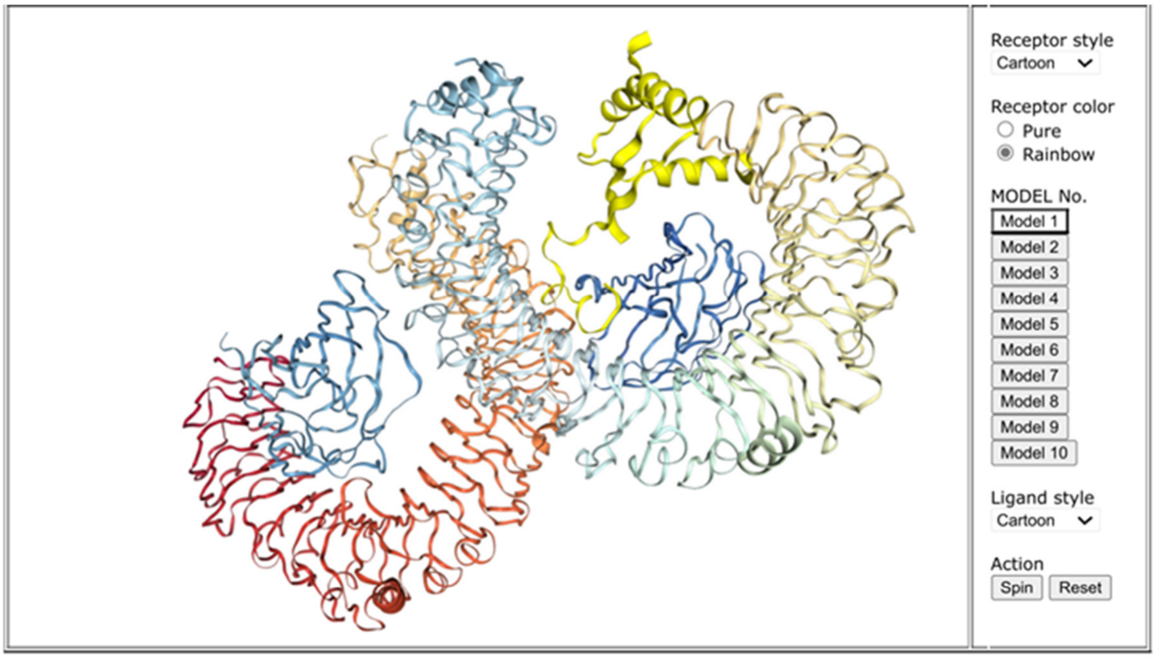

Complex Template Information (Click to Show)

Summary of the Top 10 Models

**Rank**	**1**	**2**	**3**	**4**	**5**	**6**	**7**	**8**	**9**	**10**
**Docking Score**	-284.96	-282.14	-280.66	-277.15	-267.82	-266.55	-265.67	-264.53	-264.49	-262.13
**Confidence Score**	0.9370	0.9336	0.9317	0.9271	0.9134	0.9114	0.9100	0.9081	0.9080	0.9040
**Ligand rmsd (Å)**	255.61	256.27	321.89	317.60	318.01	322.56	346.28	255.41	328.41	317.14
**Interface residues**	model_1	model_2	model_3	model_4	model_5	model_6	model_7	model_8	model_9	model_10
